# Weight loss and blood pressure lowering with GLP-1 receptor agonists and multi-hormone receptor modulators in overweight and obesity: a systematic review and meta-regression of phase 3 clinical trials

**DOI:** 10.1016/j.eclinm.2026.104251

**Published:** 2026-10-12

**Authors:** Massimo Nardone, Luxcia Kugathasan, Lauren Daunt, Ethan J. Parikh, Hao-Yuan River Zhang, Sachin V. Pasricha, Ani Orchanian-Cheff, David Z.I. Cherney, Ayodele Odutayo, Marcel H.A. Muskiet

**Affiliations:** a Division of Nephrology, Department of Medicine, University Health Network, 585 University Avenue, Toronto, Ontario M5G 2N2, Canada; b Division of Nephrology, Department of Medicine, Sunnybrook Health Sciences Centre, 2075 Bayview Avenue, Toronto, Ontario M4N 3M5, Canada; c Novo Nordisk Canada Inc, Mississauga, Ontario L5N 6M1, Canada; d Department of Medicine, University of Toronto, 6 Queen’s Park Crescent West, Toronto M5S 3H2, Canada; e Library and Information Services, University Health Network, 585 University Avenue, Toronto, Ontario M5G 2N2, Canada; f Division of Endocrinology, Department of Internal Medicine, Leiden University Medical Center, Albinusdreef 2, Leiden 2333 ZG, the Netherlands

**Keywords:** Weight loss, Blood pressure lowering, Obesity, GLP-1 receptor agonists, Multi-hormone receptor modulators

## Abstract

**Background:**

Obesity and hypertension frequently coexist, and weight-reduction is recommended as a core strategy for blood pressure (BP) management in adults with overweight or obesity. Glucagon-like peptide-1 receptor agonists (GLP-1RAs) and emerging multi-hormone receptor modulators (MHRMs) induce substantial weight loss alongside modest BP reductions. However, the extent to which weight-loss is associated with BP lowering across these therapies remains unclear.

**Methods:**

We conducted a systematic review and meta-analysis of phase 3 randomized controlled trials evaluating GLP-1RAs and MHRMs in adults with overweight or obesity (PROSPERO: CRD420251146441). We searched Ovid MEDLINE, Ovid Embase, the Cochrane Database of Systematic Reviews, and the Cochrane Central Register of Controlled Trials from 1946 to December 2025. Risk of bias was assessed using the revised Cochrane Risk of Bias tool. Placebo-adjusted changes in body weight and BP at each trial’s primary endpoint were pooled using random-effects meta-analysis with inverse-variance weighting. Meta-regression assessed the association between weight loss and BP reduction.

**Findings:**

Thirty-seven trials including 50,171 participants were analysed. Thirty-six trials had low risk of bias across all domains. Across all trials, 50% of participants were females, 16% had type 2 diabetes and 57% had hypertension. Median treatment duration was 68 weeks. Mean placebo-adjusted weight loss was −10.1% (95% CI −11.3 to −9.0) accompanied by a −4.9 mmHg (95% CI −5.5 to −4.3) reduction in systolic BP. Between-trial meta-regression showed that 76% of the variance in systolic BP reduction was explained by differences in weight loss (R^2^ = 0.76; *P* < 0.01), corresponding to a reduction of 0.37 mmHg (95% CI 0.28 to 0.45) per 1% change in body weight.

**Interpretation:**

Across phase 3 trials of GLP-1RAs and MHRMs, greater weight loss was strongly associated with larger reductions in BP, supporting pharmacological weight-loss therapies as an adjunct strategy for BP management in obesity.

**Funding:**

None.

**Research in context**

**Evidence before this study**

We reviewed previous systematic reviews and meta-analyses evaluating the influence of weight loss on blood pressure (BP) outcomes. A key meta-analysis published in *Hypertension* in 2003 by Neter et al. searched Cab Abstract, Current Contents, FSTA, and MEDLINE from inception to 2002 for randomized controlled trials evaluating intentional weight loss through energy restriction, increased physical activity, or both. Trials included adult participants with overweight or obesity, with or without hypertension, and were not restricted by geographic region. Of 97 identified articles, 25 randomized controlled trials (34 strata), comprising 4874 participants, met inclusion criteria. Risk of bias was low to moderate, with most trials using randomized parallel designs and clearly reported BP measurements. This meta-analysis demonstrated that an average net weight reduction of 5.1 kg was associated with a −4.44 mmHg (95% CI: −5.93 to −2.95) reduction in systolic BP and −3.57 mmHg (95% CI: −4.88 to −2.25) reduction in diastolic BP. Expressed per kilogram of weight loss, BP reductions were −1.05 mmHg systolic and −0.92 mmHg diastolic. Larger reductions were observed in populations achieving >5 kg weight loss or in participants taking antihypertensive medications. These associations are supported by more recent evidence. A 2023 meta-analysis by Yang et al. reported clinically meaningful reductions in systolic (−5.79 mmHg) and diastolic BP (−3.36 mmHg) following BMI reduction in adults with overweight or obesity. Consistent with the broader evidence linking weight loss to BP reduction, meta-analyses, of glucagon-like peptide receptor agonists (GLP-1RAs), including River et al. (Diabetes Obes Metab., 2024) and Wong et al. (Hypertension, 2025) have demonstrated modest reductions in BP of approximately 1–4 mmHg, with modestly greater effect sizes in patients without diabetes. However, these studies primarily reported overall treatment effects and did not quantify the relationship between the magnitude of weight loss and BP reduction. Also, much evidence predated the development of contemporary pharmacotherapies such as GLP-1RAs at obesity-specific doses and multi-hormone receptor modulators (MHRMs), which achieve greater weight loss and may exert additional weight-*independent* effects on BP. The extent to which BP reductions observed with these therapies are associated with weight loss therefore remained unclear.

**Added value of this study**

Our meta-analysis included 37 phase 3 randomized controlled trials of GLP-1RAs and MHRMs conducted in the context of obesity registration programmes, encompassing 50,171 adults with overweight or obesity, with or without comorbidities. We quantified the relationship between weight loss and systolic BP reduction, finding that approximately 76% of the between-trial variance in BP lowering was explained by differences in weight loss, corresponding to a reduction of 0.37 mmHg for each percent change in body weight. This study builds on prior evidence on the relationship between GLP-1RA and MHRM related weight loss and BP reduction by quantifying the association in the context of contemporary therapies within this class that are capable of achieving substantially greater weight loss (10–20% weight loss) and greater absolute reductions in BP in adults with overweight and obesity.

**Implications of all the available evidence**

Taken together with prior evidence, these findings reinforce weight loss as a key strategy for BP management in individuals with overweight or obesity. Contemporary pharmacotherapies that achieve larger and more sustained weight reductions can produce clinically relevant BP effects that may approach those of conventional antihypertensive medications, underscoring the clinical utility of weight-centric approaches for managing hypertension in this population. Similar to the SELECT trial, future research should examine the long-term cardiovascular outcomes of combining emerging weight-loss pharmacotherapies with traditional antihypertensive strategies and further explore the contribution of weight-independent mechanisms to BP reduction.

## Introduction

Obesity and hypertension are converging public health crises, with over half of U.S. adults with hypertension also living with obesity.^1^ Despite decades of therapeutic advances, achieving meaningful BP reduction in this population remains challenging. Consistent epidemiological and physiological evidence supports targeting obesity as an integral strategy towards BP management, as reflected in most international Hypertension Guidelines.^2^^,^^3^ Accordingly, the 2025 BP guidelines issued a Class 1 recommendation endorsing weight-loss interventions (targeting ≥5% weight reduction or ≥3 kg/m^2^ reduction in body mass index [BMI]) as a core approach for adults with overweight or obesity.^4^

First-line non-pharmacological interventions (i.e., dietary modification, structured exercise, and behavioural therapy) can reduce body weight by approximately 5–6% of initial body weight and lower systolic BP by ∼1 mmHg per kilogram lost.^5^, ^6^, ^7^ However, these interventions are often limited by challenges in long-term adherence, and the independent contributions of weight loss versus other intervention effects on BP remain difficult to disentangle. Pharmacological agents including amphetamine derivatives and bupropion/naltrexone can induce 5–9% weight loss but have been associated with psychiatric and cardiovascular risks, limiting their use, particularly in patients with hypertension.^8^^,^^9^ These limitations highlight the need for therapies that achieve substantial, durable weight loss alongside cardiometabolic improvement.

Glucagon-like peptide-1 receptor agonists (GLP-1RAs) have expanded the therapeutic options for obesity management. Originally developed for glucose-lowering in type 2 diabetes (T2D), GLP-1RAs at obesity-specific doses produce substantial weight loss (10–15%), and modest reductions in systolic BP (2–3 mmHg), with effects varying by agents, baseline BP, and diabetes status.^10^ These findings support the Class 2B recommendation for their use as adjunctive therapy to lower BP in adults with hypertension who are overweight (BMI ≥27 kg/m^2^) or obese.^4^ However, the relationship between weight loss and BP reduction remains incompletely understood, as GLP-1RAs may act through weight-dependent effects and weight-independent mechanisms – including natriuresis, improved vascular function, and autonomic modulation.^11^

This complexity is further amplified by the emergence of multi-hormone receptor modulators (MHRMs)^12^ – which represent an important advance in the treatment of T2D and obesity. These therapies typically combine central GLP-1R-agonism with activation of other hormone receptors, such as the glucose-dependent insulinotropic polypeptide (GIP), glucagon, and amylin receptor. These MHRMs can achieve mean weight-loss of ≥15%,^13^ which increasingly approach that of bariatric surgery. Emerging evidence also shows that MHRMs may produce larger reductions in BP compared to GLP-1RAs, highlighting their potential role in BP management in obesity.^14^

Given the expanding range of pharmacotherapies for overweight and obesity, and the interplay of weight-dependent and weight-independent mechanisms, quantifying the relationship between weight loss and BP reduction is warranted. Such insights could inform antihypertensive strategies, guide clinical decision-making, and help contextualize the contribution of weight loss with contemporary weight-loss interventions to cardiovascular risk factor modification in people living with overweight or obesity. To address this gap, we conducted a meta-analysis of phase 3 randomized controlled trials of GLP-1RAs and MHRMs in adults with overweight or obesity, designed for obesity treatment rather than glycemic control, to systematically quantify the association between achieved weight loss and concomitant reductions in BP.

## Methods

### Protocol design and registration

The study protocol was developed in accordance with the Preferred Reporting Items for Systematic Reviews and Meta-analyses Protocol (PRISMA-P) guidelines^15^ and the completed systematic review was conducted and reported in accordance with the PRISMA 2020 guidelines.^16^ The protocol was registered with the International Prospective Registration of Systematic Reviews (PROSPERO ID: CRD420251146441).

### Search strategy and selection criteria

We conducted a systematic review and meta-analysis of phase 3 randomized controlled trials evaluating GLP-1RAs and MHRMs on body weight and BP. Eligible studies were phase 3, double-blind or open-label, parallel-group randomized controlled trials conducted in adults (≥18 years) with overweight or obesity, with or without comorbidities, and with a minimum treatment duration of 20 weeks. Overweight and obesity was defined as either a BMI ≥ 30 kg/m^2^, a BMI ≥ 27 kg/m^2^ with the presence of at least one weight-related comorbidity (i.e., hypertension, dyslipidemia, obstructive sleep apnea, osteoarthritis, or cardiovascular disease), or a BMI ≥ 25 kg/m^2^ for trials conducted in Asia that recruited participants of Asian descent. Trials were included irrespective of diabetes status but were required to be designed for obesity treatment rather than glycemic control. Interventions included *a priori* into the systematic review were liraglutide, semaglutide, tirzepatide, cagrilintide, cagrilintide-semaglutide (CagriSema), orforglipron, taspoglutide, survodutide, retatrutide, maridebart cafraglutide, mazdutide, and ecnoglutide.

A comprehensive search strategy was developed using database-specific subject headings and text words related to GLP-1 based therapies, obesity, and phase 3 randomized trials. The Cochrane Highly Sensitive Search Strategies for identifying randomized trials were used in MEDLINE^17^ and Embase.^18^ Searches were limited to human adults. AOC and HYRZ searched the following databases on December 11, 2025: Ovid MEDLINE, Ovid Embase, Cochrane Database of Systematic Reviews (Ovid), and Cochrane Central Register of Controlled Trials (Ovid) for trials published between January 1, 1946 and December 10, 2025. The database search strategy is further described in the Supplemental Methods. The reference lists of included publications were also searched and considered for inclusion.

### Study selection

Study selection was performed in three stages. First, two investigators (amongst LK, EJP, and LD) independently screened the titles and abstracts of all identified records against pre-defined inclusion and exclusion criteria using the Covidence Extraction Tool. Second, the full-text articles of potentially eligible studies were retrieved and assessed for final inclusion. Any discrepancies between reviewers were resolved through consensus or by arbitration with a third investigator (LK, EJP, or LD). The reasons for exclusion at the full-text stage were documented, and the entire selection process is illustrated in the PRISMA flow diagram.

### Data extraction and risk of bias assessment

Four investigators independently extracted data from the included studies, and each extraction was validated by a second investigator (amongst MN, LK, EJP, and LD). Included studies were tabulated according to intervention type, population characteristics, and reported outcomes. Studies reporting placebo-adjusted changes in body weight and in-office measurements of systolic BP were included in the analysis. Baseline characteristics, including age, sex, body weight, BMI, BP, history of diabetes and hypertension, and use of antihypertensive medications were extracted, when available. Within-group and placebo-adjusted treatment effects for weight loss (expressed as a percent change) and systolic BP (expressed in mmHg) were extracted. The primary analyses used placebo-adjusted treatment effects. As specified *a priori*, when unavailable, placebo-adjusted estimates were derived from extracted within-group changes in the active treatment and placebo arms. However, this was not required because all included studies reported placebo-adjusted treatment effects at the relevant analysis timepoints. Percent change in body weight was also calculated using absolute changes. If numerical values were not reported in the manuscript text or tables, within-group treatment effects were extracted from illustrations using WebPlotDigitizer (https://automeris.io/) from the in-trial observation period. Placebo adjusted treatment effects were subsequently calculated as: $\Delta_{PA}=\mu_{T}-\mu_{C}$, where ΔPA denotes the placebo-adjusted treatment effect, μT denotes the mean change from baseline in the active treatment group, and μC denotes the mean change from baseline in the placebo group. The standard error (SE) of the placebo-adjusted estimate was calculated as: $\text{SE}\left(\Delta_{PA}\right)=\sqrt{{SE}_{T}^{2}\;+\;{SE}_{C}^{2}}$, where SET and SEC denote the standard errors of the mean change in the treatment and placebo groups, respectively. The corresponding 95% confidence interval was calculated as: $\Delta_{PA}\pm 1.96\times \text{SE}\left(\Delta_{PA}\right)$.

Treatment regimen estimands were extracted for bodyweight and BP, defined as the average treatment effect of GLP-1RAs and MHRMs versus placebo in all randomized participants, regardless of treatment discontinuation and/or use of rescue medications, in accordance with each trial’s statistical analysis plan. When treatment-regimen estimands were not reported, efficacy estimands were used, defined as the average treatment effect of GLP-1RAs and MHRMs relative to placebo for all participants who had undergone randomization, if the treatment was administered as intended as per trial statistical protocol. The primary timepoint reported for each trial was used as the *a priori* timepoint. Sensitivity analyses were performed using data extracted at ∼52 weeks (range: 48–64 weeks), to account for potential heterogeneity in follow-up duration.

Methodological quality of eligible studies was appraised using the revised Cochrane Risk of Bias tool (RoB 2).^19^ The risk of bias in all included randomized controlled trials was evaluated across five domains: bias arising from the randomization process, bias due to deviations from intended interventions, bias due to missing outcome data, bias in measurement of the outcome, and bias in selection of the reported result.^19^ Risk-of-bias assessments were conducted independently by two investigators (LK and EJP) who underwent calibration and training using RoB 2 guidance prior to formal assessment.^20^ Risk-of-bias judgments were incorporated into the interpretation of findings and, where appropriate, used to inform sensitivity analyses.

### Data analysis

The co-primary outcomes were changes in body weight (difference in the percent change from baseline) and systolic BP (difference in change in mean systolic BP from baseline) with active treatment versus placebo at the trial-specific pre-defined primary timepoint. For trials reporting multiple dose arms, dose-specific estimates were first pooled within each trial using a fixed-effect meta-analysis with inverse-variance weighting to obtain a single trial-level estimate, to appropriately account for correlated dose arms within trials. These trial-level estimates were then combined across studies using a random-effects meta-analysis with inverse-variance weighting to account for between-trial heterogeneity. Statistical heterogeneity was assessed using the I^2^ statistic and τ^2^.

To examine the association between placebo-adjusted weight loss and placebo-adjusted systolic BP reduction across trials, a random-effects meta-regression was performed using trial-level effect estimates with inverse-variance weighting. Placebo-adjusted percent weight loss was specified as the continuous moderator variable, and placebo-adjusted systolic BP reduction was the dependent variable. The proportion of between-study heterogeneity explained by the moderator was quantified using the pseudo-R^2^ statistic, calculated as the proportional reduction in estimated between-study variance (τ^2^) compared with the corresponding random-effects model. As this was a trial-level meta-regression, the findings were interpreted as associations between treatment effects across studies and were not used to infer patient-level mediation or causality. The meta-regression was performed using an unadjusted model and an adjusted model (model 2) accounting for prespecified trial-level covariates, including study follow-up duration, baseline body mass index (BMI), baseline systolic BP, proportion of participants who were male, and proportion of participants with diabetes (adjusted model 1) and the addition of pharmacological agent (adjusted model 2). Statistical analyses were performed by MN and AO using R (version 4.4.2) with the tidyverse, rstatix, meta, and metafor packages. Study selection was summarised using a PRISMA flow diagram, and baseline characteristics were presented in tables. Forest plots were used to display placebo-adjusted effects on body weight and systolic BP across trials, and a fitted meta-regression was used to illustrate the relationship between weight loss and BP reduction. Exploratory subgroup analyses were performed by pharmacological class, magnitude of mean weight loss, diabetes status, and mean baseline systolic BP (<130 versus >130 mmHg).

### Role of the funding source

There was no funding source for this study.

## Results

### Systematic review

The search identified 2077 records (667 from MEDLINE, 697 from Embase, 705 from CENTRAL, and eight from Cochrane Database of Systematic Reviews) (Fig. 1). After removal of 743 duplicates and exclusion of 250 ineligible records through automated screening tools in Covidence, 1084 titles and abstracts were independently screened for eligibility. Of these, 972 were excluded and the full texts of the remaining 112 studies were sought for retrieval of detailed assessment. Sixty-five articles were not yet published, leaving 47 full-text articles to be independently evaluated for eligibility. Ten articles were excluded due to wrong/unreported outcomes (n = 2), wrong comparator (n = 3), wrong study design (n = 3), and medication discontinuation design (n = 2). In total, 37 trials met the inclusion criteria, including semaglutide (n = 18), tirzepatide (n = 7), liraglutide (n = 6), CagriSema (n = 2), orforglipron (n = 2), mazdutide (n = 1), and ecnoglutide (n = 1).

**Fig. 1:**
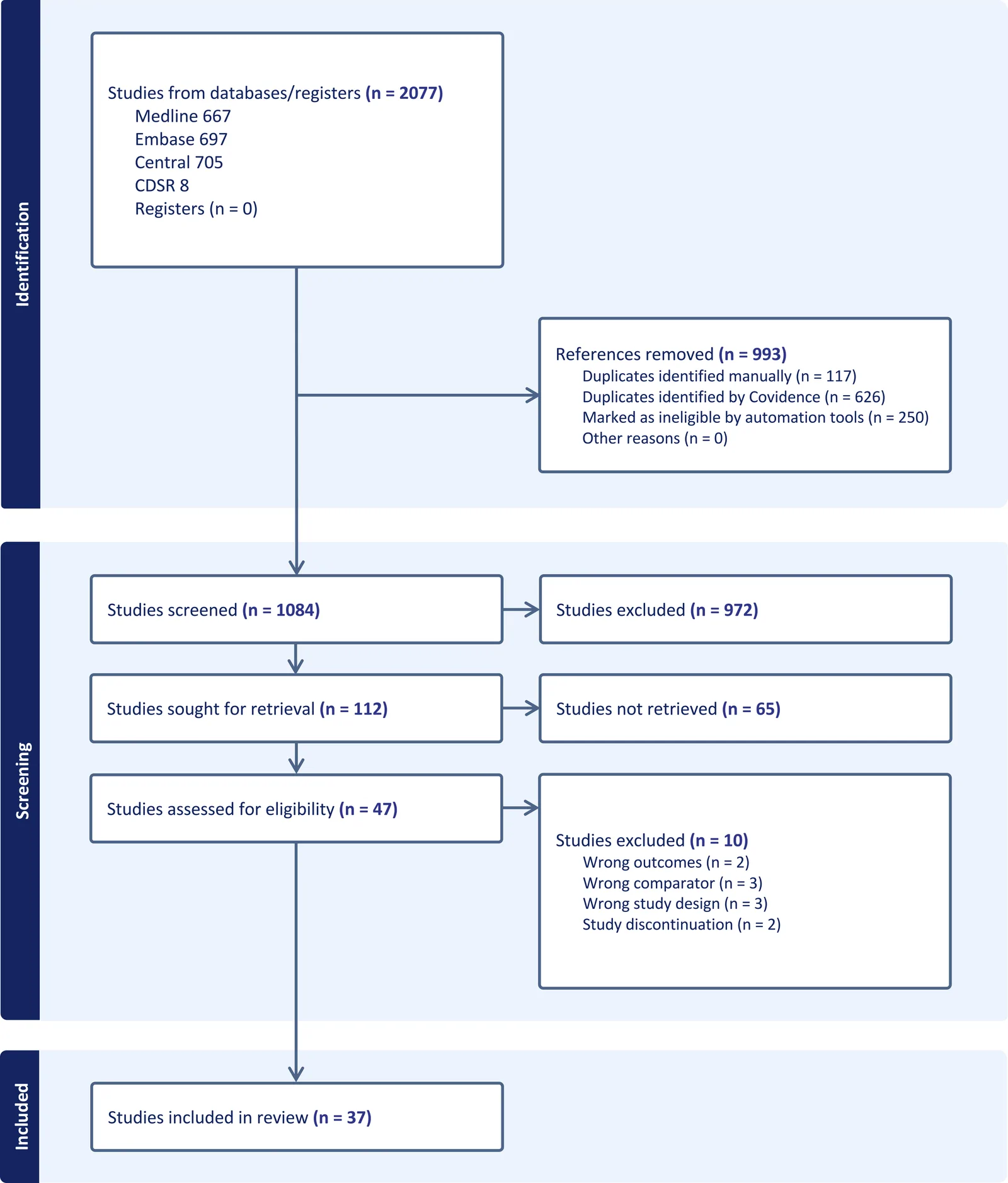
**PRISMA screening flow diagram.** CDSR, Cochrane Database of Systematic Reviews.

### Baseline characteristics

The analysis included 37 phase 3 randomized controlled clinical trials, comprising 50,171 participants with overweight or obesity. Baseline characteristics for each trial are presented in Table 1. Across all trials, 25,303 (50%) participants were female, 16% had T2D, and 28,784 (57%) of participants reported to have hypertension (reported in 33/37 trials). Median treatment duration was 68 weeks (interquartile range: 52–72).

**Table 1:** Summary of included randomized placebo-controlled trials.

Trial	Population	Agent	n	Duration	Age	Sex	Diabetes	Hypertension	Weight	BMI	Systolic BP
SCALE Maintenance (2013)	Obesity (–DM)	Liraglutide	422	56	46.2 ± 11.5	344 (81.5)	0 (0)	132 (31.3)	105.9 ± 22.2	32.8 ± 7.7	122.9 ± 12.7
SCALE Diabetes (2015)	Obesity (±DM)	Liraglutide	846	56	54.9 ± 10.5	421 (49.8)	846 (100)	586 (69.3)	105.9 ± 21.5	37.2 ± 6.7	129.4 ± 13.8
SCALE Prediabetes (2015)	Obesity (–DM)	Liraglutide	3731	56	45.1 ± 12.1	2928 (78.5)	0 (0)	1296 (34.7)	106.2 ± 21.4	38.3 ± 6.4	123.1 ± 12.9
SCALE Sleep Apnea (2016)	Obesity + OSA (–DM)	Liraglutide	359	36	48.5 ± 9.7	101 (28.1)	0 (0)	152 (42.3)	117.6 ± 24.2	39.1 ± 6.9	126.4 ± 11.9
SCALE IBT (2020)	Obesity (–DM)	Liraglutide	282	56	47.2 ± 11.5	235 (83.3)	0 (0)	–	107.6 ± 22.0	39.0 ± 7.0	126.0 ± 14.5
SCALE Insulin (2020)	Obesity (±DM)	Liraglutide	396	56	56.8 ± 10.9	207 (52.3)	396 (100)	–	99.8 ± 20.3	35.6 ± 6.2	130.5 ± 15.1
STEP-1 (2021)	Obesity (–DM)	Semaglutide	1961	68	46.3 ± 12.7	1453 (74.1)	0 (0)	706 (36)	105.3 ± 21.9	37.9 ± 6.6	126.3 ± 14.0
STEP-2 (2021)	Obesity (+DM)	Semaglutide	1210	68	55.3 ± 10.7	616 (50.9)	1210 (100)	848 (70.1)	99.8 ± 21.5	35.7 ± 6.3	130.0 ± 13.3
STEP-3 (2021)	Obesity (–DM)	Semaglutide	611	68	46.0 ± 13.0	495 (81)	0 (0)	212 (34.7)	105.8 ± 22.9	38.0 ± 6.8	124.0 ± 15.0
STEP-5 (2022)	Obesity (–DM)	Semaglutide	304	104	47.4 ± 11.0	236 (77.6)	0 (0)	118 (38.8)	106 ± 21.9	38.6 ± 6.9	125.5 ± 14.5
STEP-6 (2022)	Obesity (±DM)	Semaglutide	401	68	51.2 ± 10.8	148 (36.9)	99 (24.7)	299 (74.6)	87.5 ± 15.2	31.9 ± 4.3	133.5 ± 13.7
STEP-8 (2022)	Obesity (–DM)	Semaglutide	338	68	49.1 ± 13.2	265 (78.4)	0 (0)	142 (42)	104.5 ± 23.8	37.5 ± 6.8	124.9 ± 14.8
SURMOUNT-1 (2022)	Obesity (–DM)	Tirzepatide	2539	72	44.9 ± 12.5	1714 (67.5)	0 (0)	819 (32.3)	104.8 ± 22.1	38.0 ± 6.8	123.3 ± 12.7
OASIS-1 (2023)	Obesity (–DM)	Semaglutide	667	68	49.5 ± 12.5	485 (72.7)	0 (0)	305 (45.7)	105.3 ± 22.1	37.5 ± 6.6	129.5 ± 15.5
SELECT (2023)	Obesity (–DM)	Semaglutide	17,604	160	61.7 ± 8.9	4872 (27.7)	0 (0)	14,392 (81.8)	96.6 ± 17.7	33.3 ± 5.0	131.0 ± 15.5
STEP-HFpEF (2023)	Obesity + HFpEF (–DM)	Semaglutide	529	52	69.5 ± 9.6	297 (56.1)	0 (0)	433 (81.9)	105.0 ± 21.2	37.0 ± 5.8	132.5 ± 16.7
SURMOUNT-2 (2023)	Obesity (+DM)	Tirzepatide	938	72	54.2 ± 10.6	476 (50.7)	938 (100)	620 (66.1)	100.7 ± 21.1	36.1 ± 6.6	130.5 ± 12.1
SURMOUNT-3 (2023)	Obesity (–DM)	Tirzepatide	579	72	45.6 ± 12.2	364 (62.9)	0 (0)	199 (34.4)	101.9 ± 21.4	35.9 ± 6.3	120.9 ± 12.5
STEP-7 (2024)	Obesity (±DM)	Semaglutide	375	44	40.7 ± 11.0	170 (45.3)	96 (25.6)	172 (45.9)	96.3 ± 17.7	34.0 ± 4.8	127.0 ± 14.0
STEP-9 (2024)	Obesity + OA (–DM)	Semaglutide	407	68	56.0 ± 10.0	332 (81.6)	0 (0)	196 (48.2)	108.6 ± 24.2	40.3 ± 7.2	131.7 ± 14.3
STEP-10 (2024)	Obesity (–DM)	Semaglutide	207	52	53.0 ± 11.0	147 (71)	0 (0)	96 (46.4)	111.6 ± 22.1	40.1 ± 6.9	130.3 ± 15.0
STEP-HFpEF DM (2024)	Obesity + HFpEF (+DM)	Semaglutide	616	52	68.8 ± 8.9	273 (44.3)	616 (100)	526 (85.4)	103.7 ± 20.0	37.3 ± 5.7	134.6 ± 14.6
SURMOUNT-CN (2024)	Obesity (–DM)	Tirzepatide	210	52	32.7 ± 10.3	103 (49)	0 (0)	53 (25.2)	91.8 ± 16.0	32.3 ± 3.8	120.0 ± 12.3
SURMOUNT-OSA (2024)	Obesity + OSA (–DM)	Tirzepatide	469	52	49.9 ± 11.2	142 (30.3)	0 (0)	359 (76.5)	115.0 ± 22.9	38.8 ± 6.5	129.8 ± 12.6
STEP-11 (2025)	Obesity (–DM)	Semaglutide	150	44	39.7 ± 10.5	115 (76.7)	0 (0)	34 (22.7)	83.8 ± 18.1	31.3 ± 5.2	127.0 ± 13.0
STEP UP (2025)	Obesity (–DM)	Semaglutide	1407	72	47.0 ± 12.0	1037 (73.7)	0 (0)	585 (41.6)	113.0 ± 24.0	39.9 ± 7.1	–
STEP UP T2D (2025)	Obesity (+DM)	Semaglutide	512	72	56.8 ± 10	265 (51.8)	512 (100)	408 (79.7)	110.1 ± 22.9	38.6 ± 7.1	133.6 ± 12.6
OASIS-2 (2025)	Obesity (±DM)	Semaglutide	201	68	49.4 ± 11.2	87 (43.3)	54 (26.9)	143 (71.1)	91.9 ± 18.2	32.9 ± 4.7	134.2 ± 13.9
OASIS-4 (2025)	Obesity (±DM)	Semaglutide	307	64	47.7 ± 13.0	242 (78.8)	5 (1.6)	133 (43.3)	105.9 ± 22.3	37.6 ± 6.5	131.2 ± 16.7
REDEFINE-1 (2025)	Obesity (–DM)	CagriSema	3417	68	47.0 ± 11.8	2309 (67.6)	0 (0)	1239 (36.3)	106.9 ± 23.1	37.9 ± 6.8	127.1 ± 14.2
REDEFINE-2 (2025)	Obesity (+DM)	CagriSema	1206	68	56.1 ± 11.5	533 (44.2)	1206 (100)	885 (73.4)	102.3 ± 22.8	36.2 ± 6.8	130.2 ± 13.9
SURMOUNT-J (2025)	Obesity (–DM)	Tirzepatide	225	72	50.8 ± 10.7	92 (40.9)	0 (0)	120 (53.3)	92.0 ± 12.6	33.5 ± 4.4	125.4 ± 12.6
SUMMIT (2025)	Obesity + HFpEF	Tirzepatide	731	104	65.2 ± 10.7	393 (53.8)	352 (48.2)	–	103.0 ± 22.2	33.2 ± 8.4	128.1 ± 13.4
GLORY-1 (2025)	Obesity (–DM)	Mazdutide	610	32	34.2 ± 8.0	311 (51)	0 (0)	139 (22.8)	87.2 ± 14.0	31.1 ± 3.5	122.3 ± 11.8
SLIMMER (2025)	Obesity (–DM)	Ecnoglutide	664	40	34.2 ± 7.5	329 (49.5)	0 (0)	–	91.3 ± 16.1	32.5 ± 4.1	121.3 ± 11.5
ATTAIN-1 (2025)	Obesity (–DM)	Orforglipron	3127	72	45.1 ± 12.1	2009 (64.2)	0 (0)	1236 (39.5)	103.2 ± 22.1	36.9 ± 6.5	125.5 ± 14.6
ATTAIN-2 (2025)	Obesity (+DM)	Orforglipron	1613	72	56.8 ± 10.7	757 (46.9)	1613 (100)	1201 (74.5)	101.5 ± 22.4	35.6 ± 6.6	131.4 ± 14.3

Data are mean ± SD or n/N (%). BMI: body mass index; BP: blood pressure; DM: type 2 diabetes; HFpEF: heart failure with preserved ejection fraction; OA: Osteoarthritis; OSA; obstructive sleep apnea.

### The effect of GLP-1RAs and MHRMs on body weight

Placebo-adjusted effects of GLP-1RAs and MHRMs on body weight across trials are shown in Fig. 2. All estimates represent treatment regimen estimands. The pooled mean placebo-adjusted reduction in body weight was −10.1% (95% CI: −11.3, −9.0). Heterogeneity across studies was high (I^2^ = 98.4%, t^2^ = 13.4), reflecting variation in study populations, interventions, and dosing regimens.

**Fig. 2:**
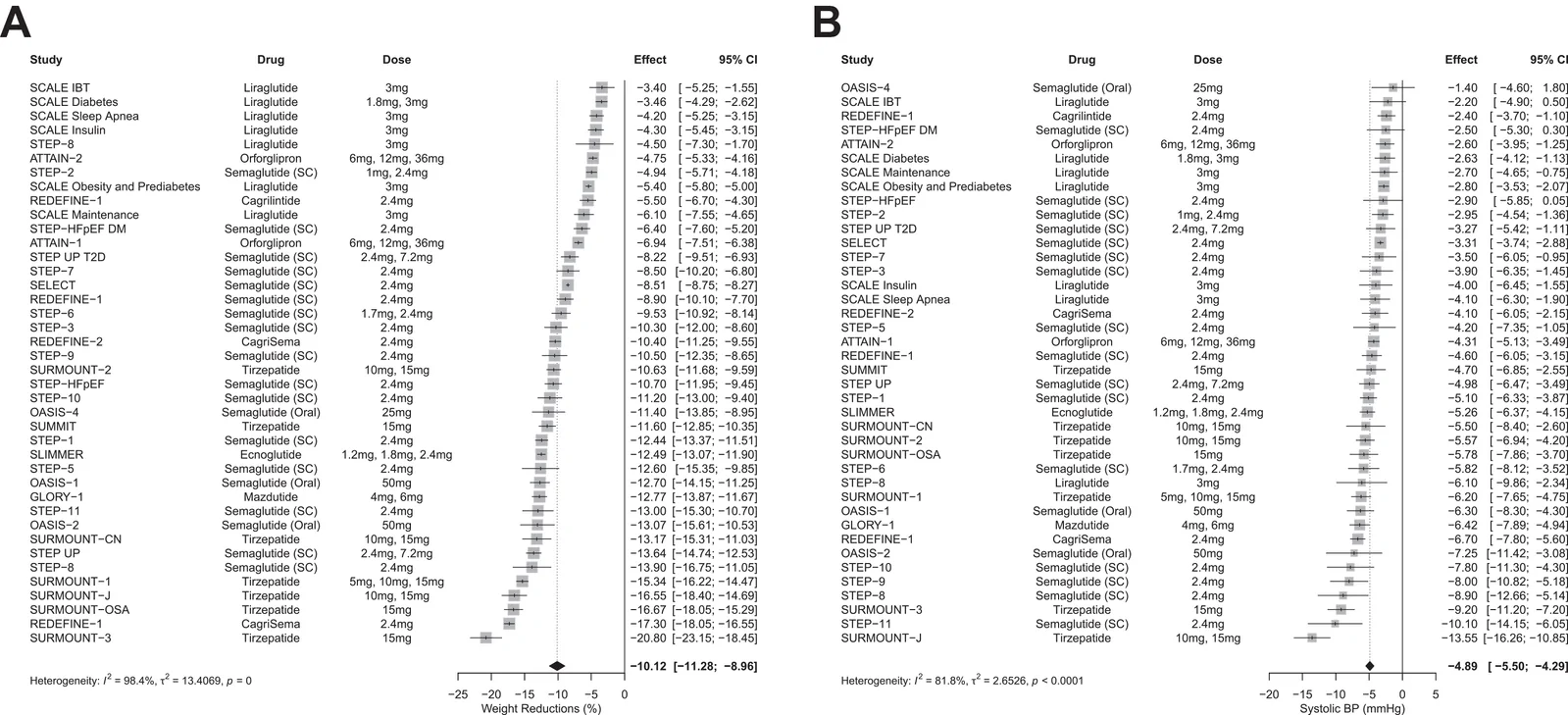
**Effect of GLP-1RAs and MHRMs on body weight and systolic blood pressure in adults with overweight or obesity.** Forest plots showing placebo-adjusted mean differences in percentage body weight change (A) and systolic blood pressure (B) across 37 phase 3 randomised controlled trials included in the meta-analysis. Squares represent study-specific estimates and horizontal lines indicate 95% CIs; square size is proportional to the inverse-variance weight of each study. Negative values indicate reductions in bodyweight or systolic blood pressure with active treatment compared with placebo.

### The effect of GLP-1RAs and MHRMs on systolic blood pressure

Placebo-adjusted effects of GLP-1RAs and MHRMs on systolic BP across trials are shown in Fig. 2. Most estimates were based on treatment regimen estimands; however, two relatively small trials (SURMOUNT-3 [n = 579] and SURMOUNT-J [n = 225]) reported only efficacy estimands, which were used accordingly. The pooled mean placebo-adjusted reduction in systolic BP was −4.9 mmHg (95% CI: −5.5, −4.3). Heterogeneity across studies was substantial (I^2^ = 81.8%, t^2^ = 2.7), reflecting the broad inclusion of a diverse population, and a variety of GLP-1RAs and MHRMs agents and doses.

### Subgroup analysis by pharmacological class

In subgroup analyses stratified by medication class, all pharmacological interventions significantly reduced systolic BP compared with placebo (Fig. 3). Tirzepatide (−7.1 mmHg [−9.0 to −5.2]) and mazdutide (−6.4 mmHg [−7.9 to −4.9]) produced the largest decreases to systolic BP. Despite achieving substantial weight loss, CagriSema and ecnoglutide were associated with smaller systolic BP reductions (−5.5 mmHg and −5.3 mmHg, respectively). Oral and subcutaneous semaglutide reduced systolic BP by 5.0 mmHg (−8.4 to −1.5) and 4.7 mmHg (−5.6 to −3.9), respectively. Estimate precision varied, with wider confidence intervals observed for oral semaglutide and CagriSema, suggesting greater uncertainty relative to other agents. Similar findings were observed for weight reduction, with greater effects seen for tirzepatide (−14.9% [−17.2 to −12.6]), CagriSema (−13.9% [−20.6 to −7.1]), mazdutide (−12.8% [−13.9 to −11.7]), and ecnoglutide (−12.5% [−13.1 to −11.9]), and more modest 4–6% reductions in body weight for liraglutide, cagrilintide, and orforglipron (Fig. 3).

**Fig. 3:**
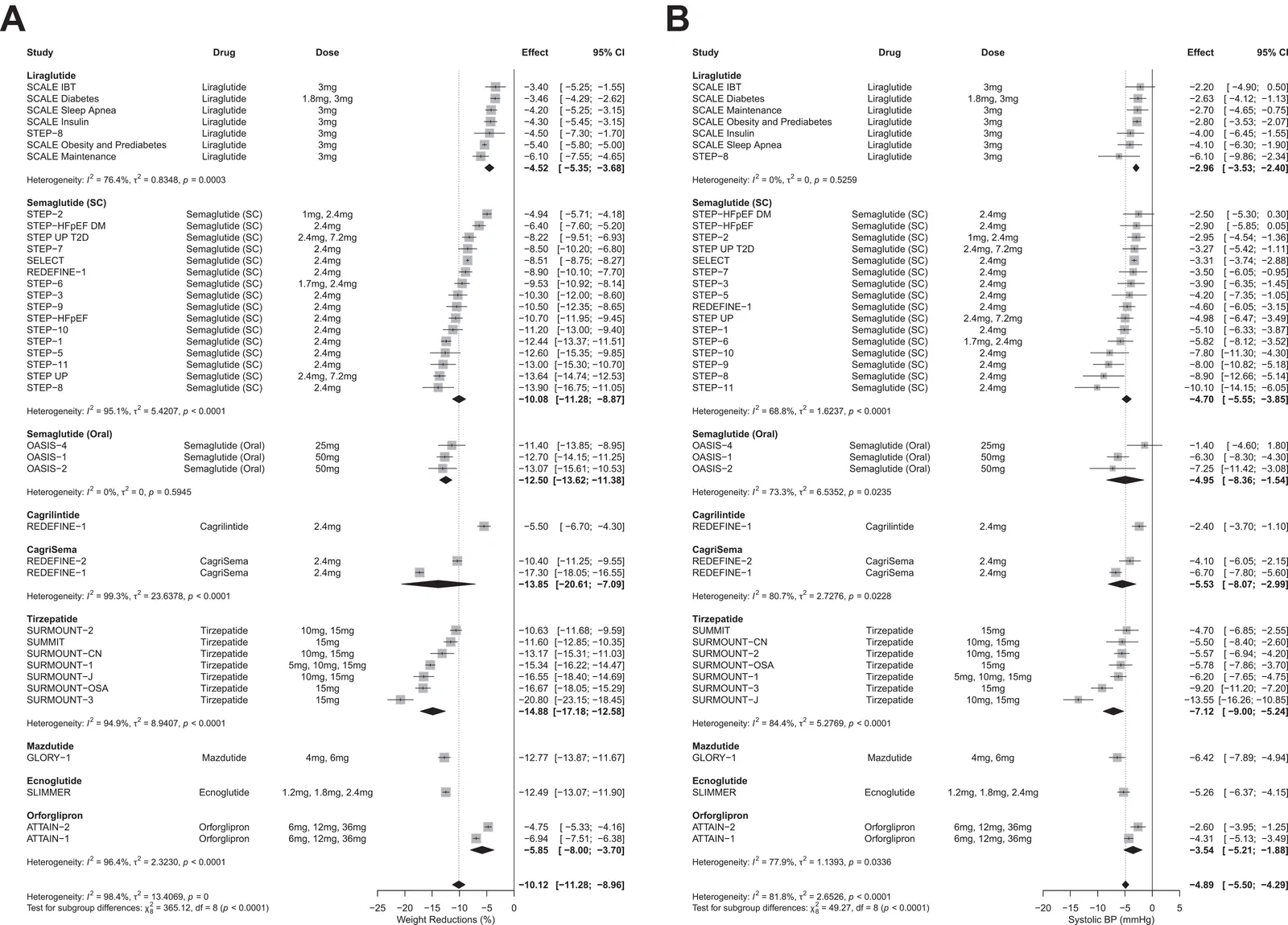
**Placebo-adjusted changes in body weight and systolic blood pressure across GLP-1RA and MHRM pharmacological interventions.** Data is presented as placebo-adjusted mean changes in body weight (A) and systolic blood pressure (B) (grey squares, expressed in mmHg), with corresponding 95% confidence intervals. The vertical dashed line represents the null effect. Negative values indicate reductions in body weight and systolic blood pressure with active treatment compared with placebo. BP, blood pressure; SC, subcutaneous.

### Subgroup analysis by magnitude of weight loss

In subgroup analyses stratified by magnitude of weight loss, we examined placebo-adjusted systolic BP reductions across predefined weight loss categories (Fig. 4). Trials achieving <5% weight loss showed a mean systolic BP reduction of −3.1 mmHg (95% CI −3.8 to −2.3), compared with −3.5 mmHg (−4.0 to −2.9) for 5–10% weight loss. Larger reductions were observed in the 10–15% subgroup (−5.4 mmHg [−6.1 to −4.8]) and were greatest among trials with >15% weight loss (−8.1 mmHg [−10.2 to −6.0]), demonstrating a graded association between increasing weight loss and greater systolic BP reduction.

**Fig. 4:**
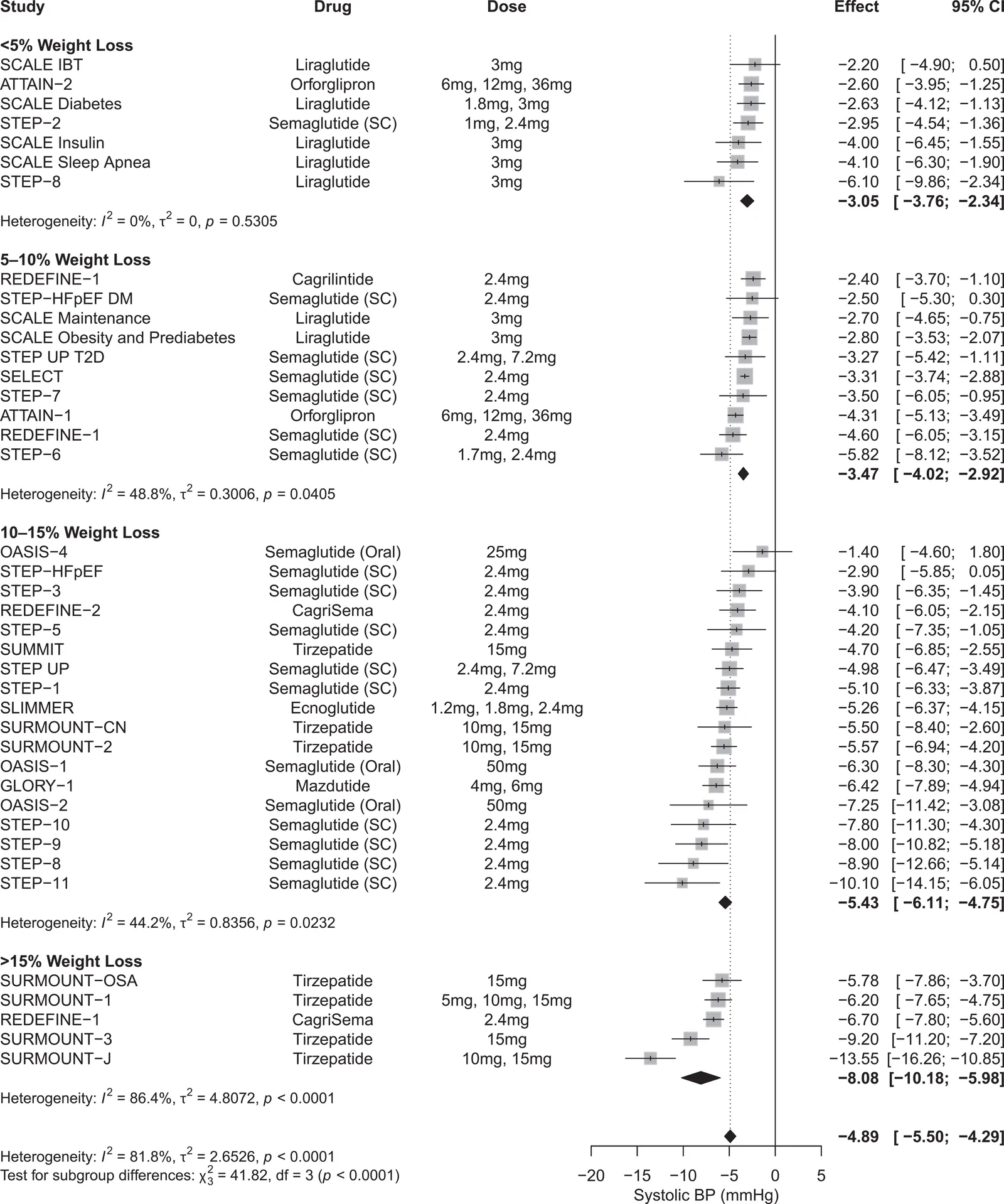
**Placebo-adjusted changes in systolic blood pressure across magnitude of weight loss.** Data is presented as placebo-adjusted mean changes in systolic blood pressure (grey squares, expressed in mmHg), with corresponding 95% confidence intervals. The vertical dashed line represents the null effect. Negative values indicate reductions in systolic blood pressure with active treatment compared with placebo. BP, blood pressure; SC, subcutaneous.

### Subgroup by diabetes status

In subgroup analyses stratified by diabetes status, GLP-1RAs and MHRMs significantly reduced both body weight and systolic BP compared with placebo in participants with and without T2D (Fig. 5). Placebo-adjusted weight loss was greater among trials enrolling participants without diabetes (−11.1% [95% CI –12.5 to −9.6]) than among those enrolling participants with diabetes (−6.6% [–8.6 to −4.7]). A similar pattern was observed for systolic BP reduction, with larger placebo-adjusted decreases in trials enrolling participants without diabetes (−5.4 mmHg [–6.2 to −4.7]) compared with those enrolling participants with diabetes (−3.5 mmHg [–4.4 to −2.6]). Despite differences in the magnitude of treatment effects, the direction of effect was consistent across both subgroups, indicating that GLP-1RAs and MHRMs lower body weight and systolic BP irrespective of diabetes status.

**Fig. 5:**
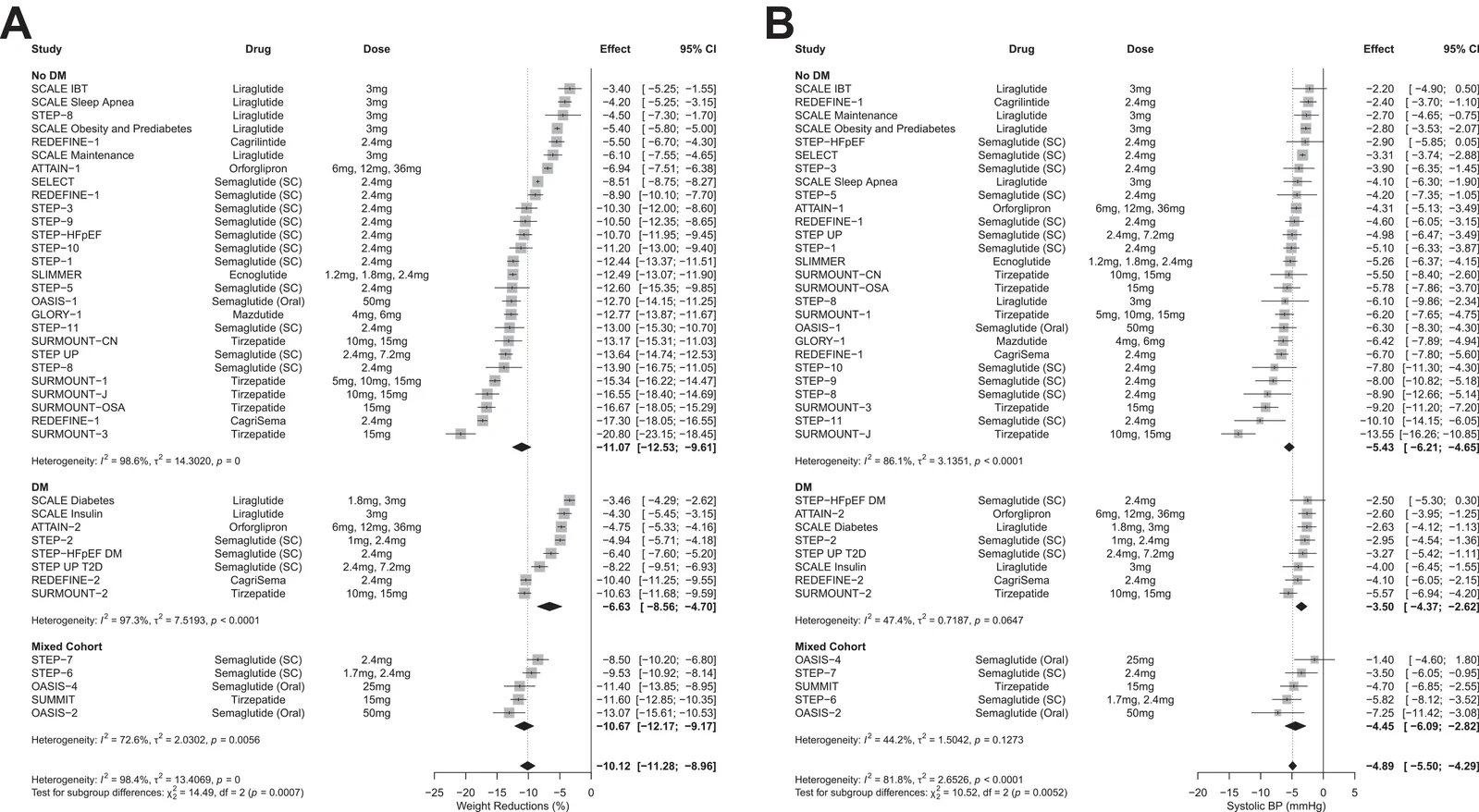
**Placebo-adjusted changes in body weight and systolic blood pressure across diabetes status.** Data is presented as placebo-adjusted mean changes in body weight (A) or systolic blood pressure (B) (grey squares, expressed as percentage change), with corresponding 95% confidence intervals. The vertical dashed line represents the null effect. Negative values indicate reductions in body weight or systolic blood pressure with active treatment compared with placebo. BP, blood pressure; SC, subcutaneous.

### Subgroup by baseline systolic blood pressure

In subgroup analyses stratified by baseline systolic BP, GLP-1RAs and MHRMs significantly reduced both body weight and systolic BP across all baseline BP categories (Fig. 6). Placebo-adjusted weight loss was similar across baseline systolic BP strata, with −10.7% (95% CI −12.6 to −8.8) in participants with baseline systolic BP < 130 mmHg and −10.0% (95% CI −11.2 to −8.9) in those with baseline systolic BP >130 mmHg. A similar relationship was observed for systolic BP reduction across baseline BP categories, with placebo-adjusted reductions of −5.3 mmHg (95% CI −6.1 to −4.4) and −4.1 mmHg (95% CI −5.0 to −3.3) for baseline systolic BP < 130 and >130 mmHg, respectively. Despite similar BP response, the direction of effect remained consistent across both subgroups, indicating robust BP-lowering effects across the full spectrum of baseline systolic BP.

**Fig. 6:**
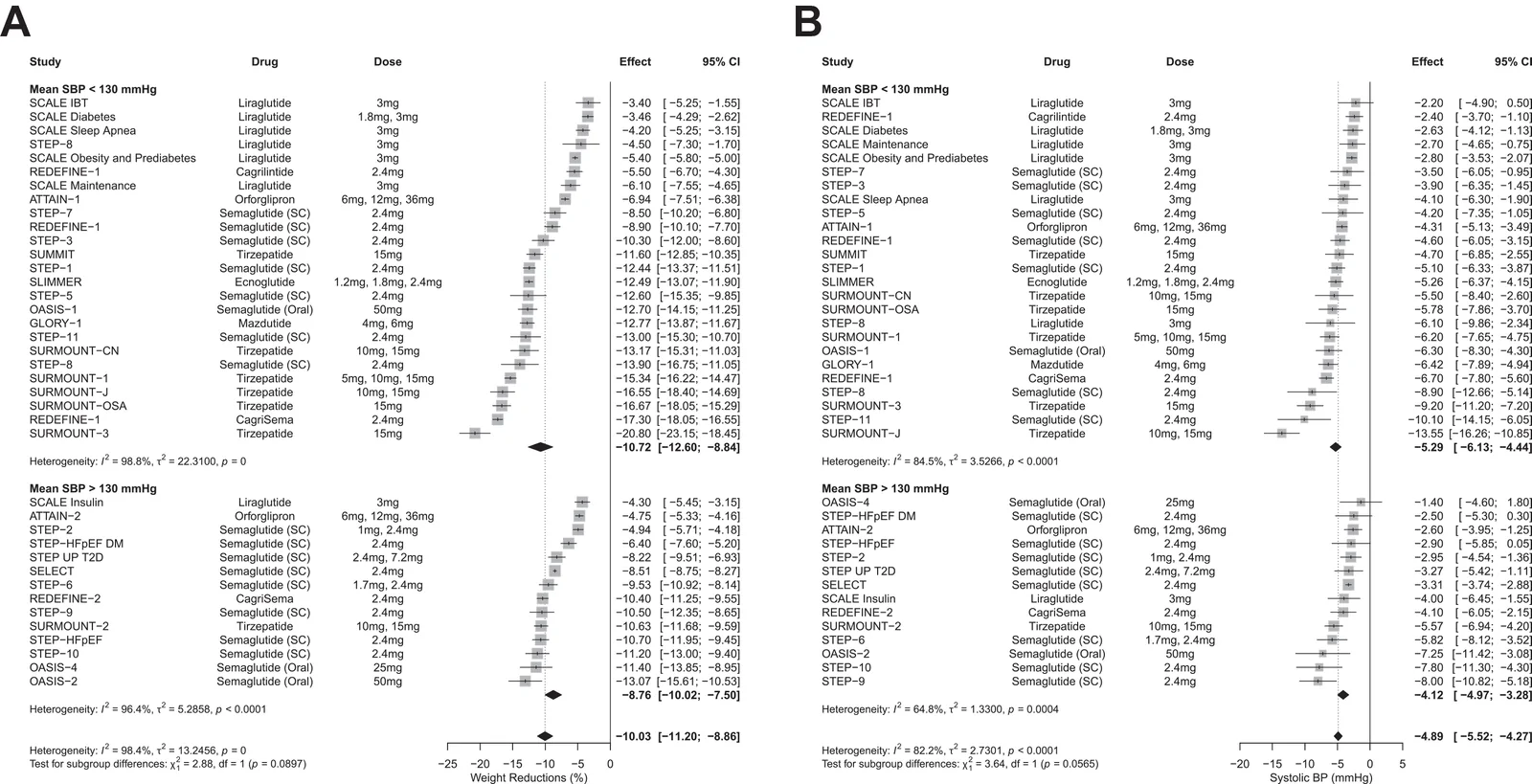
**Placebo-adjusted changes in body weight and systolic blood pressure across baseline systolic blood pressure categories.** Data is presented as placebo-adjusted mean changes in body weight (A) and systolic blood pressure (B) (grey squares, expressed in mmHg), with corresponding 95% confidence intervals. The vertical dashed line represents the null effect. Negative values indicate reductions in body weight and systolic blood pressure with active treatment compared with placebo. BP, blood pressure; SC, subcutaneous.

### Meta-regression analysis

The unadjusted meta-regression analysis (Fig. 7) showed that 76% of the between-trial variance in systolic BP lowering was explained by differences in weight loss with GLP-1RAs and MHRMs (R^2^: 0.76; *P* < 0.01). This corresponded to a reduction of 0.37 mmHg (95% CI: 0.28, 0.45) in systolic BP per % weight loss. This association remained consistent after adjusting for study follow-up duration, baseline BMI, baseline systolic BP, sex, and diabetes status (adjusted model 1: 0.37 [95% CI: 0.26, 0.48] mmHg per % weight loss), but increased with the addition of pharmacological agent (adjusted model 2: 0.52 [95% CI: 0.21, 0.82] mmHg per % weight loss).

**Fig. 7:**
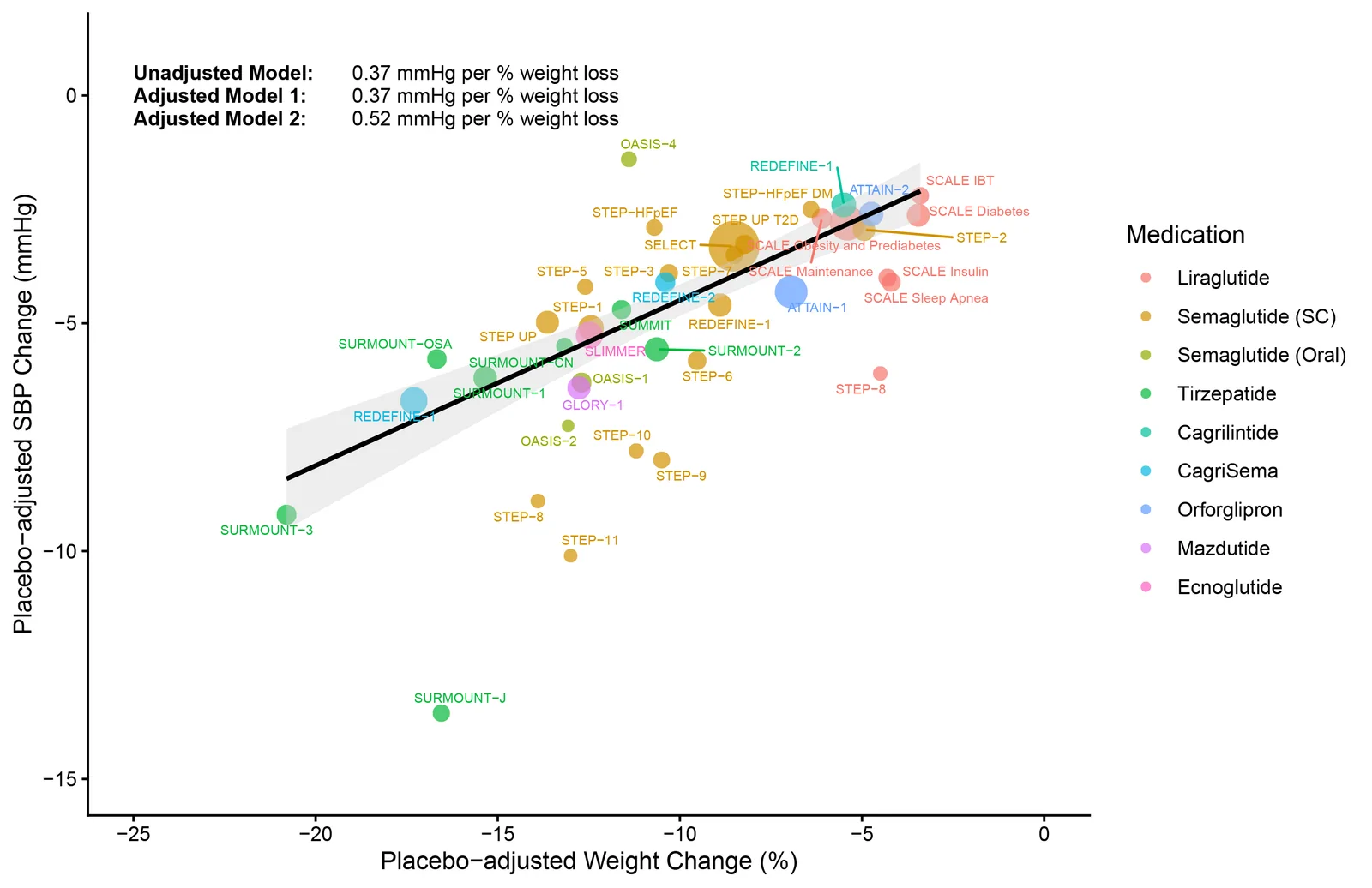
**Relationship between percent body weight change and systolic blood pressure reduction with GLP-1RA and MHRMs.** Meta-analysis of 37 phase 3 clinical trials of GLP-1RA and MHRMs in adults with overweight and obesity. Each point represents the placebo-adjusted mean change in body weight and systolic blood pressure for a single trial, with point size proportional to study weight. The solid line depicts the fitted meta-regression (β = 0.37 mmHg systolic blood pressure reduction per 1% weight loss; R^2^ = 0.76; *P* < 0.01), and the shaded area shows the 95% confidence interval. SBP, systolic blood pressure; SC, subcutaneous.

### Risk of bias assessment

The risk of bias in each study is shown in eTable S1 and eFig. S1 and S2. Thirty-six (97%) of 37 trials were assessed as having low risk of bias across all domains. Notably, the SURMOUNT-3 trial was judged at high risk of bias for incomplete outcome data because of dropout rates exceeding 10% and imbalance between groups. Sensitivity analyses excluding SURMOUNT-3 yielded results that were materially unchanged from the primary weight loss–BP meta-regression (R^2^: 0.72; *P* < 0.01), corresponded to a reduction of 0.36 mmHg (95% CI: 0.26, 0.46) in systolic BP per 1% weight loss (eFig. S3).

## Discussion

In this meta-analysis of 37 phase-3 randomized controlled trials, including more than 50,000 participants with overweight or obesity, greater weight-loss with GLP-1-based therapies was consistently associated with larger reductions in systolic BP. On average, a reduction of ∼10% in body weight corresponded to a decrease of ∼5 mmHg in systolic BP, with larger reductions observed in trials achieving ≥15% weight loss. Furthermore, trial-level meta-regression revealed that 76% of the between-trial variance in systolic BP lowering was explained by differences in weight loss, corresponding to an estimated 0.37 mmHg decrease in systolic BP per 1% weight loss. This finding is consistent with prior individual participant-level mediation analyses from SURMOUNT-1, in which weight loss was estimated to explain 68% of the reduction in systolic BP.^21^ Collectively, these findings provide quantitative evidence linking the magnitude of pharmacologically induced weight loss to BP reduction across contemporary GLP-1-based therapies for weight management, suggesting a predominantly weight-dependent effect in adults with overweight and obesity.

These results extend previous evidence from lifestyle-based interventions, which demonstrated that modest weight loss is associated with meaningful BP reductions but were limited by smaller magnitudes of weight loss and heterogeneous interventions.^5^^,^^22^ Current international recommendations recognize excess adiposity as a key modifiable determinant of hypertension and endorse weight-loss interventions as a core component of BP management. The 2025 American Heart Association/American College of Cardiology/Obesity Society Scientific Statement acknowledges GLP-1RAs as potential adjunctive therapies for individuals with hypertension and obesity because of their ability to reduce both body weight and systolic BP.^4^ Similarly, the 2024 European Society of Hypertension guidelines highlight GLP-1RAs as preferred pharmacological options for patients with obesity and metabolic comorbidities, given their favourable cardiometabolic profile and established cardiovascular benefits.^23^

While these recommendations reflect the growing integration of obesity pharmacotherapy into BP-management, prior meta-analyses have reported relatively modest reductions in systolic BP (1.5–3.4 mmHg) with GLP-1RA in a mixed cohort (including patients with overweight or obesity, diabetes, kidney and cardiovascular disease).^10^ In contrast, contemporary GLP-1-based pharmacotherapies enable substantially greater and more sustained weight loss. The magnitude of BP reduction observed in our study (4.9 mmHg overall and 8.1 mmHg with agents achieving ≥15% weight loss) exceeds that reported in earlier meta-analyses, and approaches the lower end of the range of BP reductions achieved with standard antihypertensive therapy. For reference, a 2025 meta-analysis of over 484 randomized, double-blind, placebo-controlled trials (104,176 participants) of standard dose antihypertensive monotherapy –including renin-angiotensin system (RAS) inhibitors, beta-blockers, calcium channel blockers, and diuretics– reported an average systolic BP reduction of 8.7 mmHg.^24^ However, these data were not specific to populations with overweight or obesity and should therefore be interpreted as a general benchmark. More recent meta-analyses have confirmed that GLP-1RAs consistently lower BP, although the magnitude of effect has generally been modest. A large 2025 meta-analysis of 30 randomized trials (37,072 participants) found that GLP-1RA reduced systolic BP by approximately 3.4 mmHg and diastolic BP by 1.0 mmHg versus placebo, with effects observed across different formulations and patient subgroups.^25^ Notably, these findings reinforce that BP reductions are modest at the class level but clinically relevant and consistent across heterogeneous populations. In contrast, the contemporary phase 3 obesity trials included in our analysis achieved substantially greater weight loss and were associated with larger reductions in systolic BP, reaching 8.1 mmHg among therapies achieving ≥15% weight loss. This observation suggests that the greater and more sustained 10–20% weight loss achieved with newer GLP-1RAs and MHRMs may translate into clinically meaningful additional BP-lowering. Importantly, the association between weight loss and BP reduction is not limited to pharmacotherapy trials but is consistently observed across behavioural and mixed interventions. A separate meta-analysis similarly reported a graded relationship between BMI reduction and BP lowering, with an average systolic BP decrease of approximately 5.8 mmHg for a mean BMI reduction of 2.27 kg/m^2^, and larger effects with greater BMI reductions.^26^ The study further highlighted consistent effects across both clinic and ambulatory BP measurements, supporting the robustness of this relationship beyond office-based estimates. More recent syntheses also suggest that GLP-1RA–associated BP reductions may be partially weight-loss mediated but not entirely explained by it, with additional proposed mechanisms including natriuresis and improved vascular function, supporting a pleiotropic cardiovascular profile of this drug class. Based on the meta-regression from our analysis, an estimated ∼23% placebo-adjusted weight loss (95% CI: 17–26%) would be required to achieve a similar magnitude of BP reduction.^24^ Although direct comparisons should be interpreted cautiously, these findings suggest that weight loss induced by modern pharmacotherapies may contribute meaningfully to BP control and narrow the gap with conventional antihypertensive strategies in adults with overweight and obesity.

Bariatric surgery provides an additional benchmark for interpreting these findings. Surgical interventions typically achieve 20–30% or greater weight loss and are associated with substantial reductions in BP, including improved hypertension control and remission in some individuals. Although direct comparisons should be interpreted cautiously given differences in study populations and designs, the magnitude of weight loss achieved with newer GLP-1-based therapies increasingly approaches that observed with some bariatric procedures, suggesting that these pharmacotherapies may offer clinically meaningful benefits for BP management in individuals with overweight or obesity who are not candidates for surgery or prefer non-surgical treatment approaches.

The observed association between weight loss and BP reduction suggests that weight-dependent mechanisms are likely a major contributor to the antihypertensive effects of GLP-1-based therapies. Reductions in adiposity may lower BP through multiple pathways, including improved insulin sensitivity, reduced sympathetic activation, enhanced natriuresis, and favourable vascular effects. Accordingly, therapies that achieve greater magnitudes of weight loss would be expected to produce proportionally larger reductions in BP.^22^^,^^26^ However, accumulating evidence indicates that GLP-1RAs and MHRMs may also exert weight-independent effects, including direct renal and vascular actions.^11^ These observations suggest that part of the antihypertensive effect may occur through pathways independent of weight loss. While receptor-specific mechanisms may contribute to BP regulation, their relative importance remains uncertain. For example, long-term BP effects attributable specifically to GIP receptor agonism have not been clearly established, and phase 2 studies of the amylin analogue cagrilintide did not demonstrate significant BP reductions despite meaningful weight loss. Consequently, the extent to which receptor-specific pathways contribute independently of weight loss remains to be determined in dedicated mechanistic and long-term clinical studies. Mechanistic studies have, for instance, demonstrated GLP-1–mediated natriuresis, improvements in endothelial function, reductions in arterial stiffness, and modulation of sympathetic nervous system activity.^11^ Conversely, not all GLP-1RAs appear to exert comparable effects on body weight or BP. For example, albiglutide was associated with cardiovascular benefit despite minimal weight loss and little apparent BP reduction,^27^ suggesting that cardiovascular and hemodynamic effects may not be entirely mediated through weight loss alone and may vary according to agent-specific pharmacology. In addition, emerging data suggest that certain hormonal pathways targeted by MHRMs may interact with neurohormonal systems involved in BP regulation. For example, amylin receptor agonism has been hypothesized to directly modulate the RAS, with potential effects on vascular tone and sodium handling, and possible (beneficial) interactions with RAS inhibitors.^28^ Nevertheless, the relative contribution of these weight-independent mechanisms cannot be determined from trial-level analyses and remains uncertain, particularly with newer MHRMs that engage multiple hormonal signalling pathways with potential hemodynamic effects. It therefore remains unclear whether the BP-lowering effects of emerging therapies will be driven predominantly by greater weight loss or whether receptor-specific mechanisms (such as those involving GIP, glucagon, or amylin signalling) will contribute independently. Conversely, certain receptor interactions could theoretically attenuate BP reductions achieved through weight-loss via direct vasopressor or other hypertensive effects. Several next-generation therapies are currently in phase 3 clinical development for overweight and obesity, including maridebart cafraglutide (NCT06858839), survodutide (NCT06066515), and retatrutide (NCT06859268). In addition, further phase 3 trials are ongoing for agents such as orforglipron (NCT06972459), which was included in the present meta-analysis. These studies will further clarify the relationship between weight loss and BP reduction and help define the role of MHRMs in BP management in this population.

An important additional consideration is that the magnitude of BP reduction observed in the present analysis may underestimate the true treatment potential of these agents. Several included trials reported reductions in background antihypertensive therapy among participants receiving active treatment, which would attenuate between-group differences in BP during follow-up.^29^^,^^30^ Supporting this, a participant-level analysis of three STEP trials, showed two-fold higher rates of antihypertensive de-escalation in individuals receiving semaglutide compared with controls. Mediation analyses also demonstrated that ∼89% of the observed systolic BP reduction was mediated by weight loss, with a modest but significant correlation between weight change and BP reduction (r = 0.20).^31^ Furthermore, in a secondary analysis of REDEFINE-1, ∼40% of participants on a background antihypertensive medication receiving CagriSema either de-escalated or stopped concomitant antihypertensive medications. Thus, de-escalation of concomitant antihypertensive therapy in participants receiving GLP-1RA or MHRM may attenuate the observed between-group differences in BP in randomized trials, thereby underestimating the intrinsic BP lowering effects of GLP-1-based therapies.

Substantial heterogeneity was observed across studies, likely reflecting differences in pharmacological class, dosing regimens, treatment duration, and achieved magnitude of weight loss. Our subgroup and meta-regression analyses suggest that the primary driver of this heterogeneity is treatment intensity, as reflected by differences in weight loss, with pharmacological class contributing to variation in effect size. In contrast, baseline systolic BP did not materially modify either weight loss or BP responses, indicating that baseline BP is unlikely to be a major source of heterogeneity. Some differences were observed by diabetes status, with smaller reductions in both weight and systolic BP in participants with T2D compared with those without diabetes, consistent with differences in metabolic phenotype and concomitant therapies. These findings differ from earlier evidence from the LEAD 1–6 programme, which suggested possible attenuation of BP effects at lower baseline systolic BP (“floor effect”); however, our results do not support meaningful effect modification by baseline BP. While subgroup findings from individual trials such as SURMOUNT-1 generally show consistent within-trial effects, the between-study heterogeneity observed here likely reflects differences in intervention intensity rather than true within-population effect modification. Despite this heterogeneity, the direction of effect remained consistent across all subgroups, supporting the robustness of the overall findings.

This study has several strengths, including the inclusion of a large number of phase 3 randomized controlled trials comprising more than 50,000 participants across diverse populations, enhancing generalizability. Unlike previous meta-analyses that largely included heterogeneous interventions or earlier-generation therapies with more modest weight loss effects, this analysis focused exclusively on contemporary phase 3 obesity trials of GLP-1RA and MHRM capable of producing substantial weight loss. The use of standardized placebo-adjusted estimates and meta-regression enabled quantification of the association between weight loss and systolic BP reduction across therapies with differing mechanisms and efficacy. However, several limitations should be considered. First, analyses were based on trial-level data, limiting the ability to fully adjust for individual-level confounders or explore heterogeneity in treatment response. Second, BP was not a primary endpoint in these trials. Consequently, the findings should be interpreted in the context of trials that were not specifically designed or powered to evaluate BP effects. Third, BP measurements were predominantly based on in-office assessments across trials, which may be subject to measurement variability, and may not fully reflect out-of-office BP (e.g., home BP or ambulatory BP monitoring). Fourth, the meta-regression was based on aggregate trial-level data and is therefore susceptible to ecological bias. Consequently, the observed association between weight loss and BP reduction should not be interpreted as evidence of individual-level association between weight loss and BP. Fifth, information on background antihypertensive therapy varied across trials (e.g., de-escalation during follow-up) and could not be uniformly adjusted for at the individual participant level. Although this may have influenced observed absolute BP changes, the consistency of the weight loss–BP association across sensitivity analyses suggests this is unlikely to have materially affected the main findings. Finally, variation in estimand definitions across trials may have introduced additional heterogeneity, although sensitivity analyses suggested consistent findings.

In conclusion, across phase 3 trials of GLP-1RAs and MHRMs, greater weight loss was consistently associated with larger reductions in systolic BP. These findings highlight the central role of weight reduction in BP management and support the use of contemporary obesity pharmacotherapies as part of an integrated strategy for cardiovascular risk reduction. Future studies using individual participant-level data, dedicated mechanistic investigations of weight-dependent and weight-independent pathways, and long-term cardiovascular outcomes will be essential to clarify causal mechanisms, evaluate potential interactions with antihypertensive therapies, and optimize treatment strategies.

## Contributors

MN, AO, MHAM conceived the meta-analysis and developed its protocol. AOC and HYRZ conducted the database search. LK, LD, EJP performed the abstract/text screening. MN, LK, LD, EJP performed data extraction. LK and EJP performed the risk of bias assessments. MN analysed the raw data, with validation by AO. MN, LK, and MHAM wrote the first draft of the manuscript. All authors contributed to the data interpretation and manuscript review and approved the final version of the manuscript. All authors had full access to all the data in the study. All authors had final responsibility for the decision to submit for publication.

## Data sharing statement

All data used in this meta-analysis were derived from previously published studies and are publicly available. The study protocol is accessible online via PROSPERO (CRD420251146441), and the data extraction template is provided in Appendix. Analytic code can be made available upon reasonable request to the corresponding author (MHAM).

## Declaration of interests

This paper has not been published previously in whole or part. MN is currently an employee of Novo Nordisk Canada Inc. The work presented here was conceived, conducted, analysed, interpreted, and prepared during his employment at the University Health Network and Sunnybrook Health Sciences Centre, prior to and independently of his subsequent employment with Novo Nordisk Canada Inc. Novo Nordisk had no involvement in the research and provided no funding, data, analytical support, scientific input, or assistance with manuscript preparation. AO has received research grant funding from Novo Nordisk and AstraZeneca. AO reports consultancy with Amgen Canada and honoraria from Bayer Canada for presentations. DZIC has received research grant funding from AstraZeneca, Boehringer Ingelheim/Eli Lilly, CSL Behring, Janssen, Merck, Novo Nordisk, Sanofi, and Bayer. DZIC has received consulting fees from Boehringer Ingelheim/Eli Lilly, Merck, AstraZeneca, Sanofi, Mitsubishi-Tanabe, Abbvie, Janssen, AMGEN, Bayer, Prometic, BMS, Maze, Gilead, CSL-Behring, Otsuka, Novartis, Youngene, Lexicon, Inversago, GSK, Biobridge, Vantage, Altimmune, and Novo-Nordisk. DZIC has received payment or honoraria for lectures and presentations from AstraZeneca, Bayer, Boehringer Ingelheim/Eli Lilly, Janssen, Merck, Mitsubishi Tanabe, Novo Nordisk, and Sanofi. DZIC has received support for travel to and attendance at conferences and local meetings from AstraZeneca, Bayer, Boehringer Ingelheim/Eli Lilly, Janssen, Merck, and Novo Nordisk. DZIC has received drugs from AstraZeneca. MHAM reports speaker-consultant and advisory board member for AstraZeneca, Bayer, Boehringer Ingelheim, Eli Lilly, Novo Nordisk, and Sanofi; all honoraria paid to his employer. SVP has received grant or fellowship funding from the Royal College of Physicians and Surgeons of Canada (Detweiler Travelling Fellowship) and the PSI Foundation (Educational Fellowship Funding). SVP reports consultancy with Goodself Inc. and AstraZeneca, and unrestricted educational funding from Medtronic. SVP has received travel support from Boehringer Ingelheim for conference attendance. LK, EJP, LD, HYRZ and AOC have no conflicts to disclose.

## Appendix A Supplementary data

eFigs. S1–S3 and eTable S1
