# Supplementary material for: Weight loss and blood pressure lowering with GLP-1 receptor agonists and multi-hormone receptor modulators in overweight and obesity: a systematic review and meta-regression of phase 3 clinical trials
Source: eClinicalMedicine. 2026 Oct 12;101:104251. doi: 10.1016/j.eclinm.2026.104251 (PMC13682070; doi:10.1016/j.eclinm.2026.104251)
Supplement: Supplementary file 1 [file mmc1.pdf]

SUPPLEMENTAL MATERIAL

Weight Loss and Blood Pressure Lowering with GLP-1 Receptor Agonists and Multi-Hormone Receptor Modulators in Overweight and Obesity: A Systematic Review and Meta-Regression of Phase 3 Clinical Trials

Table of Contents

Supplemental Methods ... 3

- Search Strategy for Ovid MEDLINE(R) ALL <1946 to December 10, 2025>. ... 4
- Search Strategy for Embase <1974 to 2025 December 10>. ... 5
- Search Strategy for EBM Reviews - Cochrane Central Register of Controlled Trials. ... 7
- Search Strategy for EBM Reviews - Cochrane Database of Systematic Reviews <2005 to December 10, 2025>. ... 8

Supplemental Results ... 9

- Table S1. Risk of Bias in Included Randomized Controlled Trials. ... 9
- Supplemental Figure S1: Funnel Plot for Weight ... 10
- Supplemental Figure S2: Funnel Plot for Systolic Blood Pressure ... 11
- Supplemental Figure S3: Sensitivity analysis excluding SURMOUNT-3 ... 12

Supplemental Methods

<u>Inclusion Criteria</u>

- 18 years of age or older
- Phase 3 randomized double-blind or open-label randomized controlled trial
- Overweight or obese
    - BMI ≥30 kg/m^2
    - BMI ≥27 kg/m^2 with ≥1 comorbidities
    - BMI ≥25 kg/m^2 for trials performed geographically in Asian.

<u>Exclusion Criteria</u>

- <100 participants in study
- Intervention period < 20 weeks
- Not a Phase 3 RCT
- Non-human cohort
- Non-obese cohort
- Type 1 diabetes
- Secondary analysis of an RCT

Search Strategy for Ovid MEDLINE(R) ALL <1946 to December 10, 2025>.

AOC searched the following databases on December 11, 2025: Ovid MEDLINE, Ovid Embase, Cochrane Database of Systematic Reviews (Ovid), and Cochrane Central Register of Controlled Trials (Ovid) for trials published between January 1, 1946 and December 10, 2025.

#	Searches	Results	Type	Runtime
1	exp Glucagon-Like Peptide-1 Receptor Agonists/	9538	Advanced	0.75
2	exp Glucagon-Like Peptide-1 Receptor/	4468	Advanced	0.12
3	exp Glucagon-Like Peptide 1/	13409	Advanced	0.10
4	Glucagon-Like Peptides/	3136	Advanced	0.04
5	(glp-1 or glp 1r or glp 1ra or GLP1R or GLP1RA or GLP1-RA or GLP1-R or glucagon like peptide 1 or Glucagon-like peptide receptor agonist? or incretin mimetics or Dulaglutide or Trulicity or "ly 05008" or ly 2189265 or ly05008 or ly2189265 or 923950-08-7 or Liraglutide or Victoza or Saxenda or 204656-20-2 or "4p 004" or 4p004 or 839i73s42a or nn 2211 or nn2211 or nnc 90-1170 or nnc90-1170 or rd 12014 or rd12014 or Semaglutide or Ozempic or Rybelsus or Wegovy or nn 6535 or nn 9535 or nn 9536 or nn 9924 or nn 9931 or nn 9932 or nn6535 or nn9535 or nn9536 or nn9924 or nn9931 or nn9932 or "nnc 0113 0217" or nnc01130217 or og 217 sc or og 217sc or og217sc or 910463-68-2).mp.	33352	Advanced	12.12
6	(Exenatide or Byetta or Bydureon or 141732-76-5 or 141758-74-9 or 286014-72-0 or 335149-21-8 or 9p1872d4ol or "ac 002993" or ac 2993 or ac 2993a or ac002993 or ac2993 or ac2993a or aer 601 or aer601 or da 3091 or da3091 or dance 601 or dance601 or dlp 414 or dlp414 or ex4 peptide or exenasphere or exendin 4 or ft 228 or ft228 or itca 650 or itca650 or ly 2148568 or ly2148568 or "ormd 0901" or ormd0901 or pt 302 or pt302 or Lixisenatide or Adlyxin or Adlyxine or aqve 10010 or aqve10010 or "ave 0010" or ave0010).mp.	5429	Advanced	6.47
7	(lixumiya or lyxumia or zp 10 or zp10 or 320367-13-3 or Efpeglenatide or hm 11260c or hm11260c or langlenatide or sar 439977 or sar439977 or 1296200-77-5 or Taspoglutide or bim 51077 or bim51077 or "itm 077" or itm077 or r 1583 or r1583 or ro 5073031 or ro5073031 or 275371-94-3 or Albiglutide or Albugon or Eperzan or gsk 716155 or gsk 716155a or gsk-716155 or gsk-716155a or gsk716155 or gsk716155a or naliglutide or syncria or tanzeum or 782500-75-8).mp.	377	Advanced	3.77
8	(tirzepatide or ly3298176 or zepbound or mounjaro or ly 3298176 or ly3298176 or cagrilintide or am 833 or am833 or nn 9838 or nn9838 or "nnc 0174 0833" or nnc01740833 or CagriSema or Orforglipron or ly 3502970 or ly3502970 or owl 833 or owl833 or survodutide or bi 456906 or bi456906 or ecnoglutide or "xw 004" or xw004 or retatrutide or ly 3437943 or ly3437943 or maridebart cafraglutide or amg 133 or amg133).mp.	1760	Advanced	3.12
9	or/1-8	35836	Advanced	0.04
10	exp Overweight/	302413	Advanced	0.26
11	(overweight or over-weight).mp.	108690	Advanced	2.15
12	(obesity or obese).mp.	505860	Advanced	0.62
13	or/10-12	528512	Advanced	0.19
14	9 and 13	10173	Advanced	0.06
15	randomized controlled trial.pt.	652551	Advanced	0.03
16	controlled clinical trial.pt.	95774	Advanced	0.00
17	randomized.ab.	723679	Advanced	0.00
18	placebo.ab.	265016	Advanced	0.00
19	drug therapy.fs.	2882401	Advanced	0.00
20	randomly.ab.	475889	Advanced	0.00
21	trial.ab.	791293	Advanced	0.00
22	groups.ab.	2959798	Advanced	0.01
23	15 or 16 or 17 or 18 or 19 or 20 or 21 or 22	6494697	Advanced	0.92
24	exp animals/ not humans.sh.	5404737	Advanced	0.64
25	23 not 24	5700359	Advanced	0.47
26	14 and 25	5078	Advanced	0.22
27	clinical trial, phase iii/	25939	Advanced	0.04
28	Clinical Trials, Phase III as Topic/	12359	Advanced	0.02
29	Confirmatory.mp.	54345	Advanced	0.22
30	Large-scale.mp.	211932	Advanced	0.40
31	Late-stage.mp.	35290	Advanced	0.57
32	Marketing authorization.mp.	1720	Advanced	0.18
33	Phase 3?.mp.	29484	Advanced	7.02
34	(multi-center or multi-centre or multicenter or multicentre).mp.	505372	Advanced	1.09
35	Phase III?.mp.	68548	Advanced	1.25
36	ph3?.mp.	2548	Advanced	0.36
37	phIII?.mp.	44	Advanced	0.36
38	pivotal.mp.	151753	Advanced	0.18
39	(pre-approval or preapproval).mp.	383	Advanced	0.46

40	(pre-marketing or premarketing).mp.	641	Advanced	0.27
41	(registration adj3 trial).mp.	140092	Advanced	0.39
42	or/27-41	1114859	Advanced	0.21
43	26 and 42	699	Advanced	0.05
44	limit 43 to ("all infant (birth to 23 months)" or "all child (0 to 18 years)")	66	Advanced	0.81
45	limit 43 to "all adult (19 plus years)"	389	Advanced	1.29
46	43 not 44	633	Advanced	0.01
47	45 or 46	680	Advanced	0.00
48	limit 47 to english language	672	Advanced	0.03
49	remove duplicates from 48	667	Advanced	3.01

Search Strategy for Embase <1974 to 2025 December 10>.

Conference materials were removed from Embase.

#	Searches	Results	Type	Runtime
1	exp glucagon like peptide 1 receptor agonist/	70300	Advanced	5.52
2	glucagon like peptide 1 receptor/	7277	Advanced	0.08
3	glucagon like peptide 1/	32767	Advanced	0.00
4	exp glucagon like peptide/	58524	Advanced	1.21
5	(glp-1 or glp 1r or glp 1ra or GLP1R or GLP1RA or GLP1-RA or GLP1-R or glucagon like peptide 1 or Glucagon-like peptide receptor agonist? or incretin mimetics or Dulaglutide or Trulicity or "ly 05008" or ly 2189265 or ly05008 or ly2189265 or 923950-08-7 or Liraglutide or Victoza or Saxenda or 204656-20-2 or "4p 004" or 4p004 or 839i73s42a or nn 2211 or nn2211 or nnc 90-1170 or nnc90-1170 or rd 12014 or rd12014 or Semaglutide or Ozempic or Rybelsus or Wegovy or nn 6535 or nn 9535 or nn 9536 or nn 9924 or nn 9931 or nn 9932 or nn6535 or nn9535 or nn9536 or nn9924 or nn9931 or nn9932 or "nnc 0113 0217" or nnc01130217 or og 217 sc or og 217sc or og217sc or 910463-68-2).tw,kf.	53527	Advanced	11.07
6	(Exenatide or Byetta or Bydureon or 141732-76-5 or 141758-74-9 or 286014-72-0 or 335149-21-8 or 9p1872d4ol or "ac 002993" or ac 2993 or ac 2993a or ac002993 or ac2993 or ac2993a or aer 601 or aer601 or da 3091 or da3091 or dance 601 or dance601 or dlp 414 or dlp414 or ex4 peptide or exenasphere or exendin 4 or ft 228 or ft228 or itca 650 or itca650 or ly 2148568 or ly2148568 or "ormd 0901" or ormd0901 or pt 302 or pt302 or Lixisenatide or Adlyxin or Adlyxine or aqve 10010 or aqve10010 or "ave 0010" or ave0010).tw,kf.	10159	Advanced	6.32
7	(lixumiya or lyxumia or zp 10 or zp10 or 320367-13-3 or Efpeglenatide or hm 11260c or hm11260c or langlenatide or sar 439977 or sar439977 or 1296200-77-5 or Taspoglutide or bim 51077 or bim51077 or "itm 077" or itm077 or r 1583 or r1583 or ro 5073031 or ro5073031 or 275371-94-3 or Albiglutide or Albugon or Eperzan or gsk 716155 or gsk 716155a or gsk-716155 or gsk-716155a or gsk716155 or gsk716155a or naliglutide or syncria or tanzeum or 782500-75-8).tw,kf.	1002	Advanced	3.27
8	(tirzepatide or ly3298176 or zepbound or mounjaro or ly 3298176 or ly3298176 or cagrilintide or am 833 or am833 or nn 9838 or nn9838 or "nnc 0174 0833" or nnc01740833 or CagriSema or Orforglipron or ly 3502970 or ly3502970 or owl 833 or owl833 or survodutide or bi 456906 or bi456906 or ecnoglutide or "xw 004" or xw004 or retatrutide or ly 3437943 or ly3437943 or maridebart cafraglutide or amg 133 or amg133).tw,kf.	3275	Advanced	2.69
9	or/1-8	80213	Advanced	0.06
10	exp obesity/	803150	Advanced	1.73
11	(overweight or over-weight).tw,kf.	163915	Advanced	0.87
12	(obesity or obese).tw,kf.	691527	Advanced	0.58
13	or/10-12	951554	Advanced	0.16
14	9 and 13	25923	Advanced	0.10
15	exp randomized controlled trial/	1129368	Advanced	0.12
16	controlled clinical trial/	460978	Advanced	0.04
17	random$.ti,ab.	2544904	Advanced	0.65
18	randomization/	101714	Advanced	0.01
19	intermethod comparison/	317958	Advanced	0.05
20	placebo.ti,ab.	470652	Advanced	0.13
21	(compare or compared or comparison).ti,ab.	8949605	Advanced	1.28
22	((evaluated or evaluate or evaluating or assessed or assess) and (compare or compared or comparing or comparison)).ab.	3416171	Advanced	3.00
23	(open adj label).ti,ab.	192401	Advanced	0.36
24	((double or single or doubly or singly) adj (blind or blinded or blindly)).ti,ab.	373011	Advanced	0.85
25	double blind procedure/	314253	Advanced	0.01
26	parallel group$1.ti,ab.	53911	Advanced	2.71
27	(crossover or cross over).ti,ab.	158518	Advanced	0.57
28	((assign$ or match or matched or allocation) adj5 (alternate or group$1 or intervention$1 or patient$1 or subject$1 or participant$1)).ti,ab.	522288	Advanced	12.12

29	(assigned or allocated).ti,ab.	620814	Advanced	0.09
30	(controlled adj7 (study or design or trial)).ti,ab.	649077	Advanced	2.82
31	(volunteer or volunteers).ti,ab.	325611	Advanced	0.33
32	human experiment/	737236	Advanced	0.04
33	trial.ti.	575057	Advanced	0.00
34	or/15-33	11859821	Advanced	2.36
35	(random$ adj sampl$ adj7 ("cross section$" or questionnaire$1 or survey$ or database$1)).ti,ab. not (comparative study/ or controlled study/ or randomi?ed controlled.ti,ab. or randomly assigned.ti,ab.)	10842	Advanced	5.61
36	cross-sectional study/ not (exp randomized controlled trial/ or controlled clinical trial/ or controlled study/ or randomi?ed controlled.ti,ab. or control group$1.ti,ab.)	482008	Advanced	2.26
37	(((case adj control$) and random$) not randomi?ed controlled).ti,ab.	24885	Advanced	2.29
38	systematic review.ti,ab. not (trial or study).ti.	437532	Advanced	0.80
39	(nonrandom$ not random$).ti,ab.	21548	Advanced	0.46
40	"random field$".ti,ab.	3266	Advanced	0.72
41	(random cluster adj3 sampl$).ti,ab.	1811	Advanced	0.37
42	(review.ab. and review.pt.) not trial.ti.	1354816	Advanced	0.34
43	"we searched".ab. and (review.ti. or review.pt.)	61256	Advanced	1.08
44	"update review".ab.	156	Advanced	0.19
45	(databases adj4 searched).ab.	81092	Advanced	0.05
46	(rat or rats or mouse or mice or swine or porcine or murine or sheep or lambs or pigs or piglets or rabbit or rabbits or cat or cats or dog or dogs or cattle or bovine or monkey or monkeys or trout or marmoset$1).ti. and animal experiment/	1329177	Advanced	1.46
47	animal experiment/ not (human experiment/ or human/)	2811867	Advanced	1.05
48	or/35-47	5073546	Advanced	0.38
49	34 not 48	10361856	Advanced	1.31
50	14 and 49	8127	Advanced	0.54
51	phase 3 clinical trial/	117636	Advanced	0.02
52	"phase 3 clinical trial (topic)"/	57127	Advanced	0.00
53	Confirmatory.tw,kf.	70333	Advanced	0.21
54	Large-scale.tw,kf.	247046	Advanced	0.72
55	Late-stage.tw,kf.	48725	Advanced	0.66
56	Marketing authorization.tw,kf.	3728	Advanced	0.34
57	Phase 3?.tw,kf.	85992	Advanced	10.36
58	(multi-center or multi-centre or multicenter or multicentre).tw,kf.	532450	Advanced	1.26
59	Phase III?.tw,kf.	96991	Advanced	0.69
60	ph3?.tw,kf.	3557	Advanced	0.15
61	phIII?.tw,kf.	84	Advanced	0.08
62	pivotal.tw,kf.	194317	Advanced	0.14
63	(pre-approval or preapproval).tw,kf.	834	Advanced	0.59
64	(pre-marketing or premarketing).tw,kf.	920	Advanced	0.17
65	(registration adj3 trial).tw,kf.	111195	Advanced	0.40
66	or/51-65	1367338	Advanced	0.17
67	50 and 66	1041	Advanced	0.06
68	limit 67 to (embryo <first trimester> or infant <to one year> or child <unspecified age> or preschool child <1 to 6 years> or school child <7 to 12 years> or adolescent <13 to 17 years>)	60	Advanced	0.43
69	limit 67 to (adult <18 to 64 years> or aged <65+ years>)	833	Advanced	0.31
70	67 not 68	981	Advanced	0.01
71	69 or 70	1013	Advanced	0.01
72	limit 71 to english language	1003	Advanced	0.02
73	limit 72 to (conference abstract or conference paper or "conference review" or conference proceeding)	278	Advanced	0.97
74	72 not 73	725	Advanced	0.00
75	remove duplicates from 74	697	Advanced	3.43

Search Strategy for EBM Reviews - Cochrane Central Register of Controlled Trials.

#	Searches	Results	Type	Runtime
1	exp Glucagon-Like Peptide-1 Receptor Agonists/	0	Advanced	0.00
2	exp Glucagon-Like Peptide-1 Receptor/	252	Advanced	0.01
3	exp Glucagon-Like Peptide 1/	2553	Advanced	0.01
4	Glucagon-Like Peptides/	546	Advanced	0.00
5	(glp-1 or glp 1r or glp 1ra or GLP1R or GLP1RA or GLP1-RA or GLP1-R or glucagon like peptide 1 or Glucagon-like peptide receptor agonist? or incretin mimetics or Dulaglutide or Trulicity or "ly 05008" or ly 2189265 or ly05008 or ly2189265 or 923950-08-7 or Liraglutide or Victoza or Saxenda or 204656-20-2 or "4p 004" or 4p004 or 839i73s42a or nn 2211 or nn2211 or nnc 90-1170 or nnc90-1170 or rd 12014 or rd12014 or Semaglutide or Ozempic or Rybelsus or Wegovy or nn 6535 or nn 9535 or nn 9536 or nn 9924 or nn 9931 or nn 9932 or nn6535 or nn9535 or nn9536 or nn9924 or nn9931 or nn9932 or "nnc 0113 0217" or nnc01130217 or og 217 sc or og 217sc or og217sc or 910463-68-2).mp.	9715	Advanced	0.78
6	(Exenatide or Byetta or Bydureon or 141732-76-5 or 141758-74-9 or 286014-72-0 or 335149-21-8 or 9p1872d4ol or "ac 002993" or ac 2993 or ac 2993a or ac002993 or ac2993 or ac2993a or aer 601 or aer601 or da 3091 or da3091 or dance 601 or dance601 or dlp 414 or dlp414 or ex4 peptide or exenasphere or exendin 4 or ft 228 or ft228 or itca 650 or itca650 or ly 2148568 or ly2148568 or "ormd 0901" or ormd0901 or pt 302 or pt302 or Lixisenatide or Adlyxin or Adlyxine or aqve 10010 or aqve10010 or "ave 0010" or ave0010).mp.	1794	Advanced	0.43
7	(lixumiya or lyxumia or zp 10 or zp10 or 320367-13-3 or Efpeglenatide or hm 11260c or hm11260c or langlenatide or sar 439977 or sar439977 or 1296200-77-5 or Taspoglutide or bim 51077 or bim51077 or "itm 077" or itm077 or r 1583 or r1583 or ro 5073031 or ro5073031 or 275371-94-3 or Albiglutide or Albugon or Eperzan or gsk 716155 or gsk 716155a or gsk-716155 or gsk-716155a or gsk716155 or gsk716155a or naliglutide or syncria or tanzeum or 782500-75-8).mp.	293	Advanced	0.35
8	(tirzepatide or ly3298176 or zepbound or mounjaro or ly 3298176 or ly3298176 or cagrilintide or am 833 or am833 or nn 9838 or nn9838 or "nnc 0174 0833" or nnc01740833 or CagriSema or Orforglipron or ly 3502970 or ly3502970 or owl 833 or owl833 or survodutide or bi 456906 or bi456906 or ecnoglutide or "xw 004" or xw004 or retatrutide or ly 3437943 or ly3437943 or maridebart cafraglutide or amg 133 or amg133).mp.	872	Advanced	0.17
9	or/1-8	11039	Advanced	0.01
10	exp Overweight/	26311	Advanced	0.00
11	(overweight or over-weight).mp.	24971	Advanced	0.07
12	(obesity or obese).mp.	62602	Advanced	0.04
13	or/10-12	66772	Advanced	0.01
14	9 and 13	3464	Advanced	0.01
15	clinical trial, phase iii/	0	Advanced	0.00
16	Clinical Trials, Phase III as Topic/	2844	Advanced	0.00
17	Confirmatory.mp.	4503	Advanced	0.01
18	Large-scale.mp.	9140	Advanced	0.04
19	Late-stage.mp.	1339	Advanced	0.03
20	Marketing authorization.mp.	266	Advanced	0.02
21	Phase 3?.mp.	74056	Advanced	0.76
22	(multi-center or multi-centre or multicenter or multicentre).mp.	228022	Advanced	0.09
23	Phase III?.mp.	53957	Advanced	0.08
24	ph3?.mp.	278	Advanced	0.01
25	phIII?.mp.	29	Advanced	0.00
26	pivotal.mp.	8514	Advanced	0.01
27	(pre-approval or preapproval).mp.	43	Advanced	0.04
28	(pre-marketing or premarketing).mp.	38	Advanced	0.01
29	(registration adj3 trial).mp.	87829	Advanced	0.12
30	or/15-29	380270	Advanced	0.04
31	14 and 30	705	Advanced	0.02

Search Strategy for EBM Reviews - Cochrane Database of Systematic Reviews <2005 to December 10, 2025>.

#	Searches	Results	Type	Runtime
1	(glp-1 or glp 1r or glp 1ra or GLP1R or GLP1RA or GLP1-RA or GLP1-R or glucagon like peptide 1 or Glucagon-like peptide receptor agonist? or incretin mimetics or Dulaglutide or Trulicity or "ly 05008" or ly 2189265 or ly05008 or ly2189265 or 923950-08-7 or Liraglutide or Victoza or Saxenda or 204656-20-2 or "4p 004" or 4p004 or 839i73s42a or nn 2211 or nn2211 or nnc 90-1170 or nnc90-1170 or rd 12014 or rd12014 or Semaglutide or Ozempic or Rybelsus or Wegovy or nn 6535 or nn 9535 or nn 9536 or nn 9924 or nn 9931 or nn 9932 or nn6535 or nn9535 or nn9536 or nn9924 or nn9931 or nn9932 or "nnc 0113 0217" or nnc01130217 or og 217 sc or og 217sc or og217sc or 910463-68-2).ti,ab.	22	Advanced	0.13
2	(Exenatide or Byetta or Bydureon or 141732-76-5 or 141758-74-9 or 286014-72-0 or 335149-21-8 or 9p1872d4ol or "ac 002993" or ac 2993 or ac 2993a or ac002993 or ac2993 or ac2993a or aer 601 or aer601 or da 3091 or da3091 or dance 601 or dance601 or dlp 414 or dlp414 or ex4 peptide or exenasphere or exendin 4 or ft 228 or ft228 or itca 650 or itca650 or ly 2148568 or ly2148568 or "ormd 0901" or ormd0901 or pt 302 or pt302 or Lixisenatide or Adlyxin or Adlyxine or aqve 10010 or aqve10010 or "ave 0010" or ave0010).ti,ab.	4	Advanced	0.07
3	(lixumiya or lyxumia or zp 10 or zp10 or 320367-13-3 or Efpeglenatide or hm 11260c or hm11260c or langlenatide or sar 439977 or sar439977 or 1296200-77-5 or Taspoglutide or bim 51077 or bim51077 or "itm 077" or itm077 or r 1583 or r1583 or ro 5073031 or ro5073031 or 275371-94-3 or Albiglutide or Albugon or Eperzan or gsk 716155 or gsk 716155a or gsk-716155 or gsk-716155a or gsk716155 or gsk716155a or naliglutide or syncria or tanzeum or 782500-75-8).ti,ab.	1	Advanced	0.04
4	(tirzepatide or ly3298176 or zepbound or mounjaro or ly 3298176 or ly3298176 or cagrilintide or am 833 or am833 or nn 9838 or nn9838 or "nnc 0174 0833" or nnc01740833 or CagriSema or Orforglipron or ly 3502970 or ly3502970 or owl 833 or owl833 or survodutide or bi 456906 or bi456906 or ecnoglutide or "xw 004" or xw004 or retatrutide or ly 3437943 or ly3437943 or maridebart cafraglutide or amg 133 or amg133).ti,ab.	3	Advanced	0.03
5	or/1-4	23	Advanced	0.00
6	(overweight or over-weight).ti,ab.	83	Advanced	0.02
7	(obesity or obese).ti,ab.	149	Advanced	0.01
8	6 or 7	155	Advanced	0.00
9	5 and 8	8	Advanced	0.00

Supplemental Results

Table S1. Risk of Bias in Included Randomized Controlled Trials.

Study	Random Sequence Generation	Allocation Concealment	Blinding of Participants	Blinding of Outcome Assessment	Incomplete Outcome Data	Selective Reporting	Other Bias
SCALE Maintenance (2013)	Low risk	Low risk	Low risk	Low risk	Low risk	Low risk	Low risk
SCALE Diabetes (2015)	Low risk	Low risk	Low risk	Low risk	Low risk	Low risk	Low risk
SCALE Obesity and Prediabetes (2015)	Low risk	Low risk	Low risk	Low risk	Low risk	Low risk	Low risk
SCALE Sleep Apnea (2016)	Low risk	Low risk	Low risk	Low risk	Low risk	Low risk	Low risk
SCALE IBT (2020)	Low risk	Low risk	Low risk	Low risk	Low risk	Low risk	Low risk
SCALE Insulin (2020)	Low risk	Low risk	Low risk	Low risk	Low risk	Low risk	Low risk
STEP-1 (2021)	Low risk	Low risk	Low risk	Low risk	Low risk	Low risk	Low risk
STEP-2 (2021)	Low risk	Low risk	Low risk	Low risk	Low risk	Low risk	Low risk
STEP-3 (2021)	Low risk	Low risk	Low risk	Low risk	Low risk	Low risk	Low risk
STEP-5 (2022)	Low risk	Low risk	Low risk	Low risk	Low risk	Low risk	Low risk
STEP-6 (2022)	Low risk	Low risk	Low risk	Low risk	Low risk	Low risk	Low risk
STEP-8 (2022)	Low risk	Low risk	Low risk	Low risk	Low risk	Low risk	Low risk
SURMOUNT-1 (2022)	Low risk	Low risk	Low risk	Low risk	Low risk	Low risk	Low risk
OASIS-1 (2023)	Low risk	Low risk	Low risk	Low risk	Low risk	Low risk	Low risk
SELECT (2023)	Low risk	Low risk	Low risk	Low risk	Low risk	Low risk	Low risk
STEP-HFpEF (2023)	Low risk	Low risk	Low risk	Low risk	Low risk	Low risk	Low risk
SURMOUNT-2 (2023)	Low risk	Low risk	Low risk	Low risk	Low risk	Low risk	Low risk
SURMOUNT-3 (2023)	Low risk	Low risk	Low risk	Low risk	High risk	Low risk	Low risk
STEP-7 (2024)	Low risk	Low risk	Low risk	Low risk	Low risk	Low risk	Low risk
STEP-9 (2024)	Low risk	Low risk	Low risk	Low risk	Low risk	Low risk	Low risk
STEP-10 (2024)	Low risk	Low risk	Low risk	Low risk	Low risk	Low risk	Low risk
STEP-HFpEF DM (2024)	Low risk	Low risk	Low risk	Low risk	Low risk	Low risk	Low risk
SURMOUNT-CN (2024)	Low risk	Low risk	Low risk	Low risk	Low risk	Low risk	Low risk
SURMOUNT-OSA (2024)	Low risk	Low risk	Low risk	Low risk	Low risk	Low risk	Low risk
STEP-11 (2025)	Low risk	Low risk	Low risk	Low risk	Low risk	Low risk	Low risk
STEP UP (2025)	Low risk	Low risk	Low risk	Low risk	Low risk	Low risk	Low risk
STEP UP T2D (2025)	Low risk	Low risk	Low risk	Low risk	Low risk	Low risk	Low risk
OASIS-2 (2025)	Low risk	Low risk	Low risk	Low risk	Low risk	Low risk	Low risk
OASIS-4 (2025)	Low risk	Low risk	Low risk	Low risk	Low risk	Low risk	Low risk
REDEFINE-1 (2025)	Low risk	Low risk	Low risk	Low risk	Low risk	Low risk	Low risk
REDEFINE-2 (2025)	Low risk	Low risk	Low risk	Low risk	Low risk	Low risk	Low risk
SURMOUNT-J (2025)	Low risk	Low risk	Low risk	Low risk	Low risk	Low risk	Low risk
SUMMIT (2025)	Low risk	Low risk	Low risk	Low risk	Low risk	Low risk	Low risk
GLORY-1 (2025)	Low risk	Low risk	Low risk	Low risk	Low risk	Low risk	Low risk
SLIMMER (2025)	Low risk	Low risk	Low risk	Low risk	Low risk	Low risk	Low risk
ATTAIN-1 (2025)	Low risk	Low risk	Low risk	Low risk	Low risk	Low risk	Low risk
ATTAIN-2 (2025)	Low risk	Low risk	Low risk	Low risk	Low risk	Low risk	Low risk

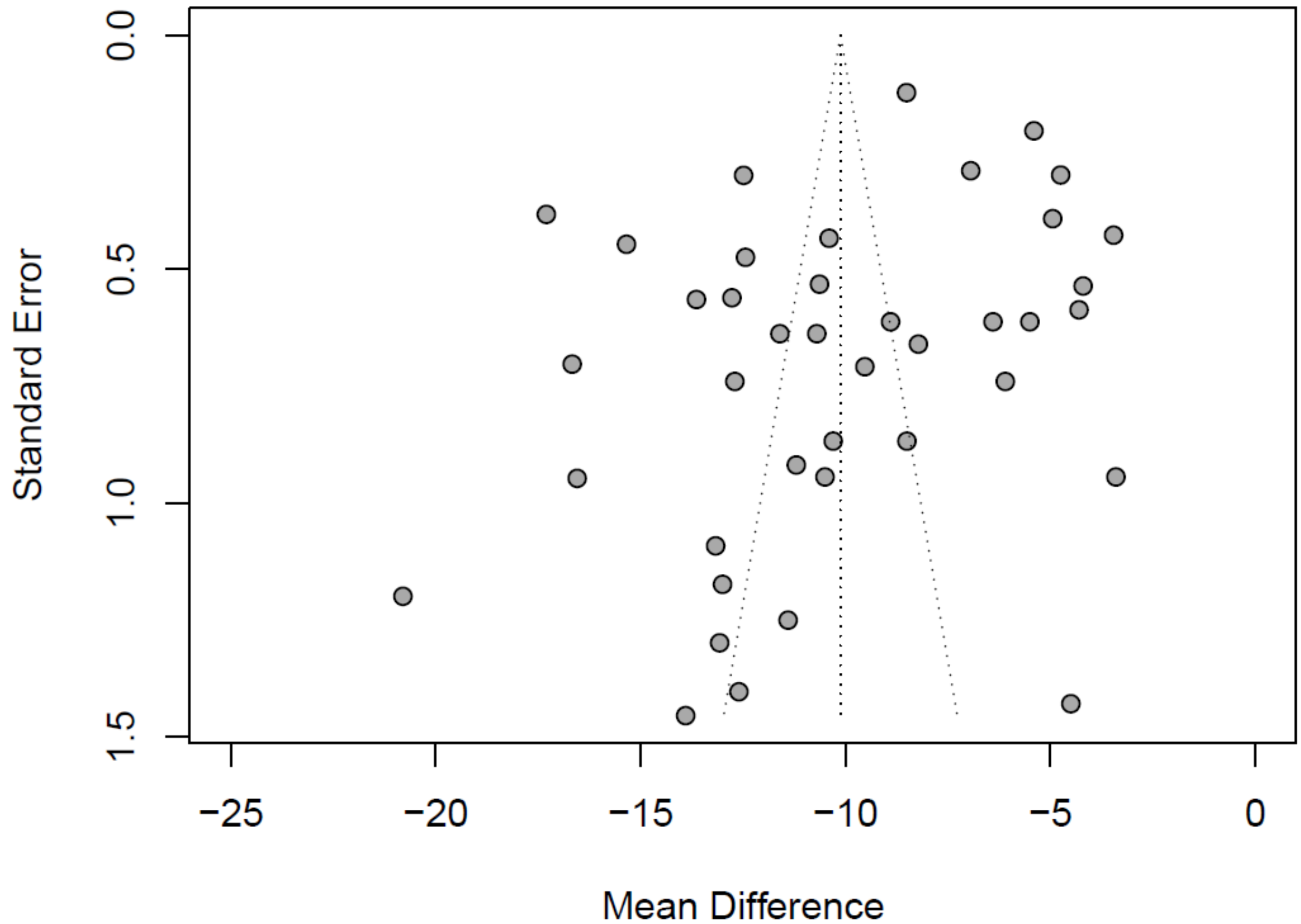

Supplemental Figure S1: Funnel Plot for Weight

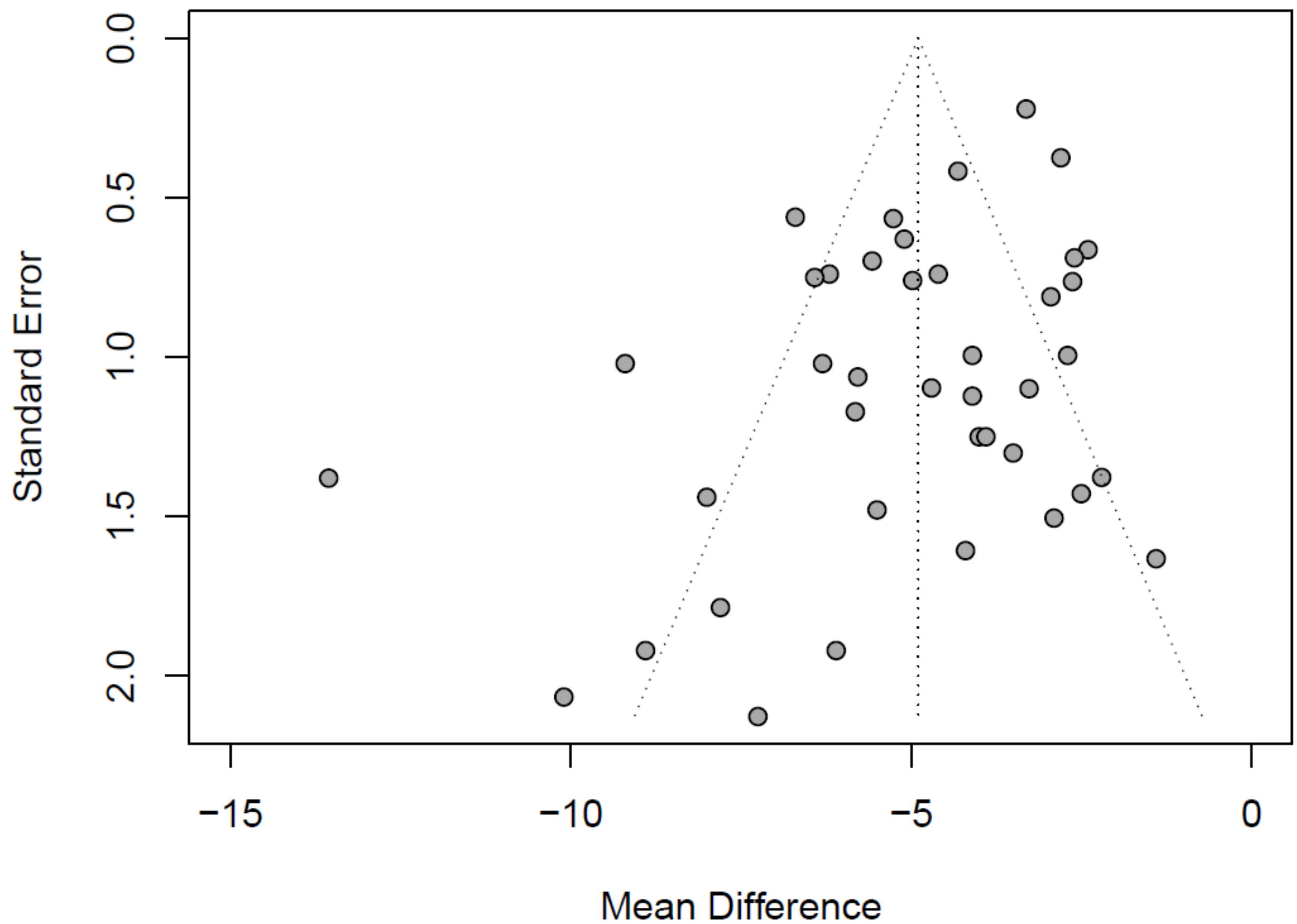

Supplemental Figure S2: Funnel Plot for Systolic Blood Pressure

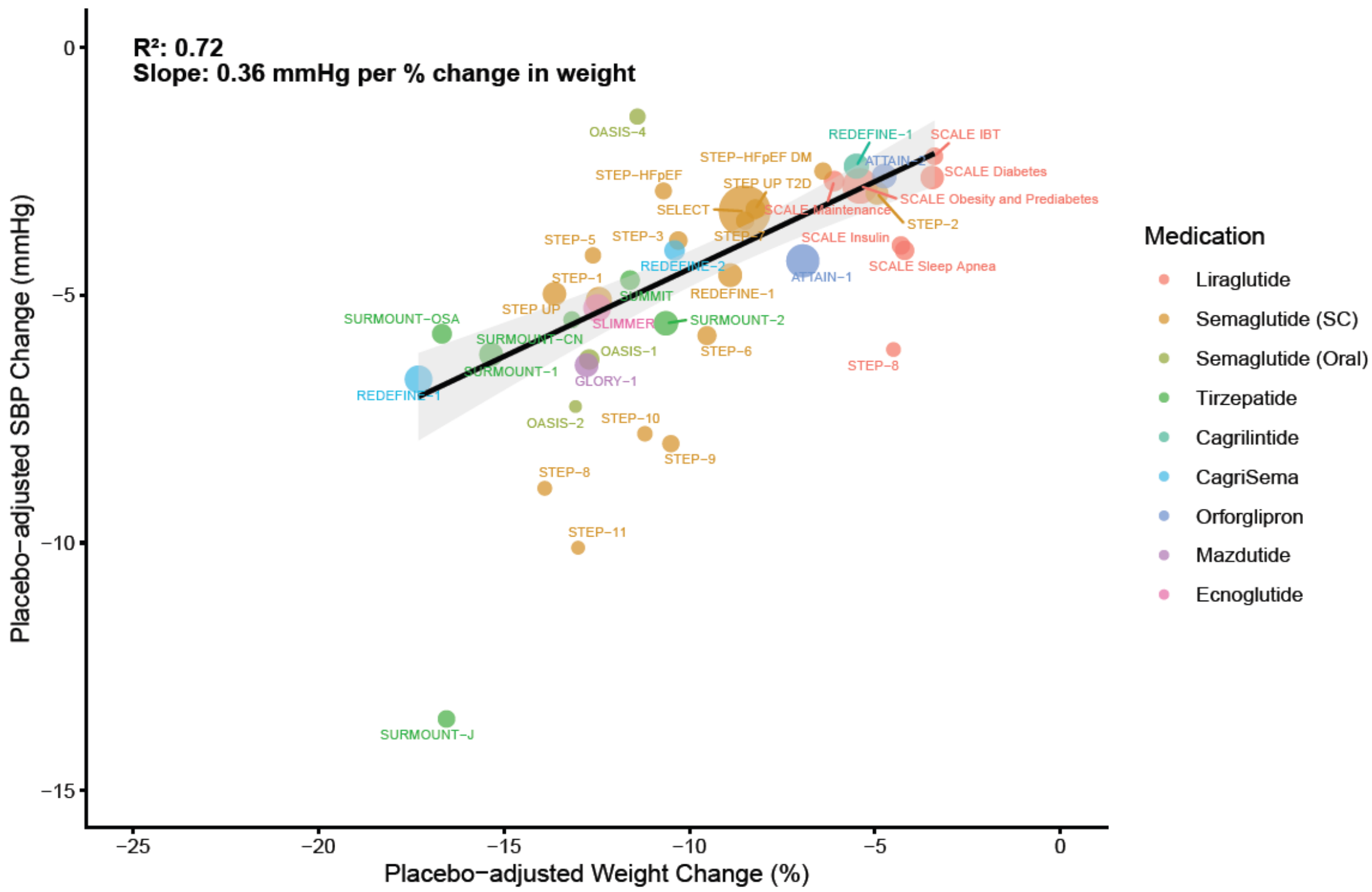

Supplemental Figure S3: Sensitivity analysis excluding SURMOUNT-3.
